# Cutaneous adverse events with antibody-drug conjugates: a FAERS-based pharmacovigilance study

**DOI:** 10.3389/fmed.2026.1847032

**Published:** 2026-05-25

**Authors:** Huiwen Sun, Jinhan Chen, Qian Xu, Chen Chen, Jinsheng Yu, Qijin Shu

**Affiliations:** The First Affiliated Hospital of Zhejiang Chinese Medical University (Zhejiang Provincial Hospital of Chinese Medicine), Hangzhou, Zhejiang, China

**Keywords:** antibody-drug conjugates, cutaneous adverse events, FAERS, pharmacovigilance, real-world study

## Abstract

**Introduction:**

Antibody-drug conjugates (ADCs) have revolutionized oncology by integrating the target specificity of monoclonal antibodies with the potency of cytotoxic payloads. However, despite a subset of clinical trials and case reports documenting cutaneous adverse events (CAEs) associated with these agents, comprehensive systematic characterization of ADC-related CAEs remains elusive.

**Methods:**

Data extracted from the FDA Adverse Event Reporting System (FAERS) spanning Q1 2004 to Q2 2025 included patient baseline characteristics. Disproportionality analyses—reporting odds ratio (ROR) and information component (IC)—were employed to identify safety signals, along with supplementary assessments of time-to-onset (TTO), clinical outcomes, and age- and gender-stratified risks.

**Results:**

A total of 3,631 CAEs were identified. Concomitantly, a set of 31 positive signals (PT) was detected at the preferred term level, encompassing common manifestations such as rash, alopecia, bullous dermatitis, generalized exfoliative dermatitis, onycholysis, spider naevus, as well as rare but severe events with robust signals including Stevens-Johnson syndrome (SJS)-toxic epidermal necrolysis (TEN) overlap and generalized exfoliative dermatitis. Notably, positive PTs exhibited marked drug specificity: enfortumab vedotin was associated with the highest number of positive signals, whereas belantamab mafodotin displayed minimal signals. The median TTO of CAEs was 15 days, with trastuzumab deruxtecan demonstrating the shortest latency. Regarding clinical outcomes, hospitalization (23.4%) and death (7.7%) were prominent. Stratified analyses further revealed that elderly patients and males were more susceptible to ADC-related CAEs.

**Conclusion:**

Our findings highlight significant risks of CAEs linked to ADCs, emphasizing the need for risk-stratified monitoring and personalized management. Furthermore, we identified some safety signals of CAEs that are not currently annotated in drug labeling, which provide critical real-world evidence to supplement and extend the limited clinical trial safety data.

## 1 Introduction

Antibody-Drug Conjugates (ADCs), a transformative class of targeted therapeutics, have reshaped treatment paradigms for multiple advanced or refractory solid tumors and hematological malignancies (1). Through the covalent linkage of monoclonal antibodies to cytotoxic payloads via chemical linkers, ADCs enable precise delivery of potent cytotoxic agents to tumor sites, minimizing off-target damage while enhancing antitumor efficacy (2, 3).

Robust evidence from pivotal phase III clinical trials confirms that ADCs confer superior survival outcomes compared to conventional cytotoxic chemotherapy. In the DESTINY-Gastric-04 trial, trastuzumab deruxtecan (T-DXd) significantly improved median overall survival (14.7 vs. 11.4 months; HR = 0.70, 95%CI: 0.55–0.90; *P* = 0.0044) and objective response rate (44.3% vs. 29.1%; *P* = 0.0006) versus ramucirumab plus paclitaxel in patients with HER2-positive advanced gastric/gastroesophageal junction adenocarcinoma (4). Additionally, in the ASCENT-03 trial, sacituzumab govitecan (SG) achieved superior progression-free survival in patients with advanced triple-negative breast cancer (median: 9.7 vs. 6.9 months; HR = 0.62, 95%CI: 0.50–0.77; *P* < 0.001) compared to standard chemotherapy (5). Driven by continuous biotechnological innovation, ADCs have undergone three generations of evolution (6), with advancements in site-specific conjugation, linker stability, and payload design optimizing their therapeutic index. As of 2025, at least 15 ADCs had received Food and Drug Administration (FDA) approval (7). These advancements collectively underscore the potential of ADCs in oncology.

Despite these therapeutic gains, ADCs remain plagued by adverse events (AEs), with hematological toxicity, ocular toxicity, and peripheral neuropathy well documented (8). Notably, the incidence of ADC-associated cutaneous adverse events (CAEs) is rising, encompassing a broad spectrum: alopecia, stomatitis, and morbilliform, bullous, and exfoliative rashes, plus rare but severe entities including Stevens-Johnson syndrome (SJS) and Toxic epidermal necrolysis (TEN) (9). A meta-analysis of 169 ADC clinical trials (22,492 patients) showed that rashes accounted for 15.5% of grade 3 AEs (10). In the EV-301 trial, enfortumab vedotin (EV) treatment was associated with a 43.1% rash incidence, including 14.6% grade ≥ 3 events (11). In DESTINY-Breast06, T-Dxd-related CAE rates were: alopecia (48.39%), dry skin (5.07%), palmar-plantar erythrodysaesthesia syndrome (0.92%), and rash (5.99%) (12). The pathogenesis underlying these CAEs may be associated with types of chemical linkers, the cytotoxic mechanisms of payloads, the expression of target antigens on keratinocytes, or even the route of subcutaneous administration. Nevertheless, the exact mechanisms remain elusive (9, 13, 14).

Owing to the paucity of comprehensive assessments of ADC-mediated cutaneous toxicity and the lack of systematic characterization in clinical trial safety evaluations, a thorough real-world surveillance data analysis is imperative. The FDA Adverse Event Reporting System (FAERS), one of the world’s largest post-marketing drug safety databases, provides a robust data source for this study. We aim to leverage its real-world data to systematically analyze the risks, temporal patterns, and outcome disparities of ADC-related CAEs, and identify previously unrecognized high-risk cutaneous adverse event signal, and summarize the distinct cutaneous toxicity patterns of the three major ADC payload classes (MMAE, PBD, DM1). This work intends to provide evidence for stratified real-world risk assessment and clinical decision-making by healthcare professionals, enabling personalized risk stratification based on payload class, patient subgroups and newly identified high-risk signals, thereby compensating for the limitations of controlled clinical trials and improving patient care quality.

## 2 Materials and methods

### 2.1 Data source and collection

**FIGURE 1:**
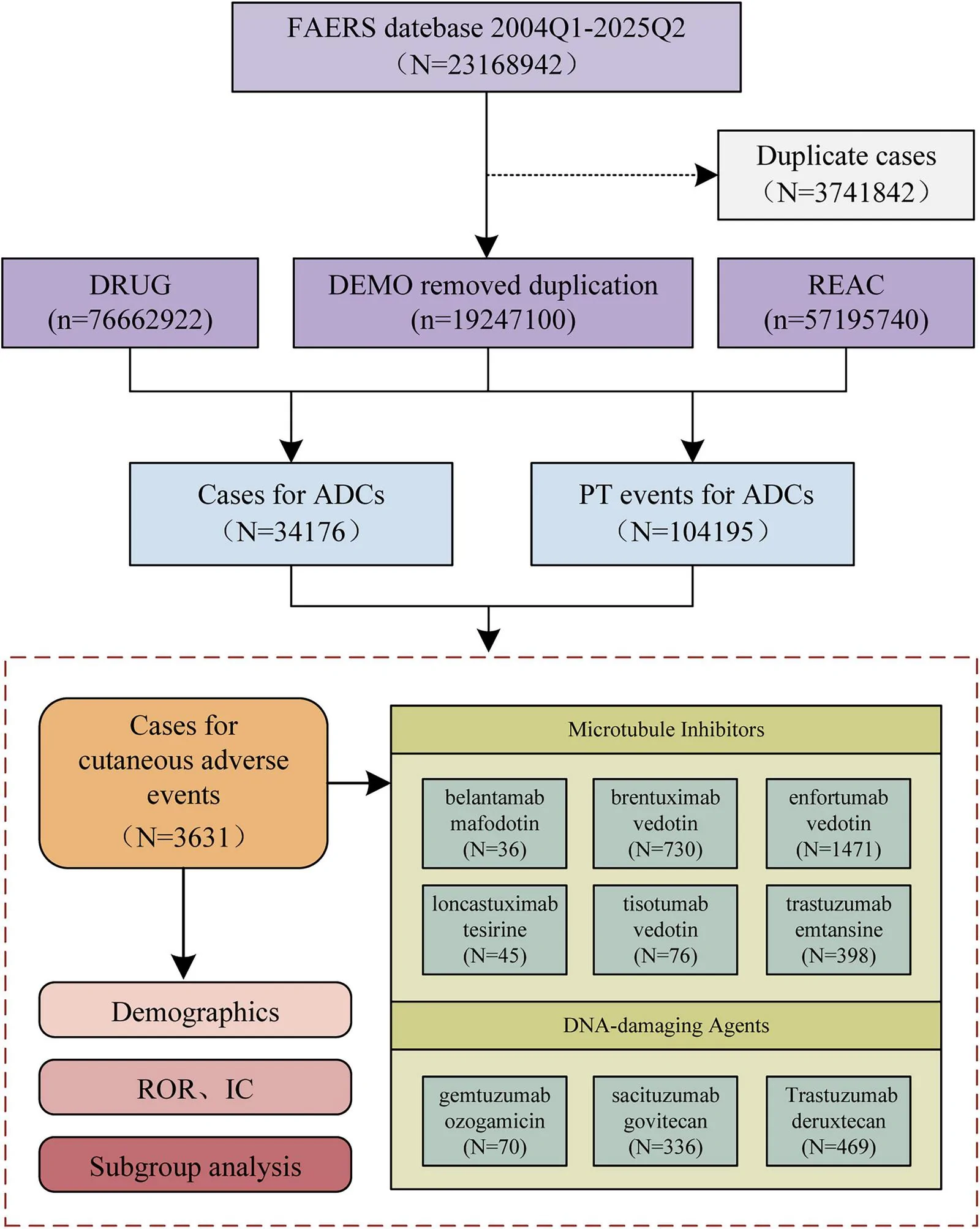
The flow diagram of the study. DEMO, demographic and administrative information; DRUG, drug information; REAC, coded AEs.

This retrospective study analyzed CAEs associated with ADCs using data from the FAERS database, which spans from the first quarter of 2004 to the second quarter of 2025 (Figure 1). All AEs were coded using Preferred Terms (PTs) from the Medical Dictionary for Regulatory Activities (MedDRA, version 27.0), which allows for further hierarchical classification into high-level terms (HLT), high-level group terms (HLGT), and system organ class (SOC). The FAERS data comprises seven dataset categories: DEMO (demographics), DRUG (medications), REAC (adverse events), OUTC (outcomes), RPSR (report sources), THER (treatment duration), and INDI (indications), which can be linked via the PRIMARYID and CASEID.

### 2.2 Data pre-processing and extraction

A total of 23,168,942 reports were retrieved. In accordance with FDA guidelines, duplicate reports were eliminated by matching the variables PRIMARYID, CASEID, and FDA_DT from the DEMO table. For the same CASEID, the latest FDA_DT was retained; for matching CASEID and FDA_DT, the largest PRIMARYID was kept, with 3,741,842 duplicates removed (Figure 1). This strategy was adopted to minimize overcounting bias, although it may carry a minor risk of underestimating the true incidence of recurrent or delayed-onset adverse events. An initial candidate pool of 15 ADCs was systematically refined to 9 FDA-approved targeted agents for the present study: belantamab mafodotin (Belamaf), brentuximab vedotin (BV), EV, gemtuzumab ozogamicin (GO), loncastuximab tesirine (Lonca), SG, tisotumab vedotin (TV), T-DXd, and trastuzumab emtansine (T-DM1). After identifying cases using all relevant drug names (active ingredients, brand names, salt forms), only reports in which an ADC was designated as the “Primary Suspect (PS)” drug were included.

### 2.3 Data mining and statistical analysis

In this study, disproportionality was assessed using the Frequentist (reporting odds ratio, ROR) (15) and Bayesian (information component, IC) (16) approaches to explore the potential association between ADCs and CAEs. Signal thresholds were defined as: a lower limit of the 95% confidence interval (CI) for ROR (ROR025) > 1 or a lower limit of the 95% CI for IC (IC025) > 0 with at least 3 reports. These thresholds indicate that the reporting frequency of the target drug-AE pair was higher than that in the control group. Additionally, time to onset, defined as the time interval from ADC administration to CAE occurrence, was also assessed, and the median time (in days) along with its corresponding interquartile range (IQR) was presented. Furthermore, the Weibull distribution model was used for dynamic analysis of the temporal evolution of AE occurrence; this model is characterized by two core parameters: scale (α) and shape (β). To optimize prognostic assessment of CAEs (17). Furthermore, the Weibull distribution model was used for dynamic analysis of the temporal evolution of AE occurrence; we also analyzed the proportional distributions of clinical outcomes across different ADC regimens. Moreover, we conducted age- and gender-stratified subgroup analyses via a two-way stratified disproportionality analysis to reduce the impact of confounding variables (18). Statistical analyses were conducted using SAS version 9.4, while data visualization was performed with R version 4.0.2 and GraphPad Prism version 10.6.1.

## 3 Results

### 3.1 Clinical characteristics

**FIGURE 2:**
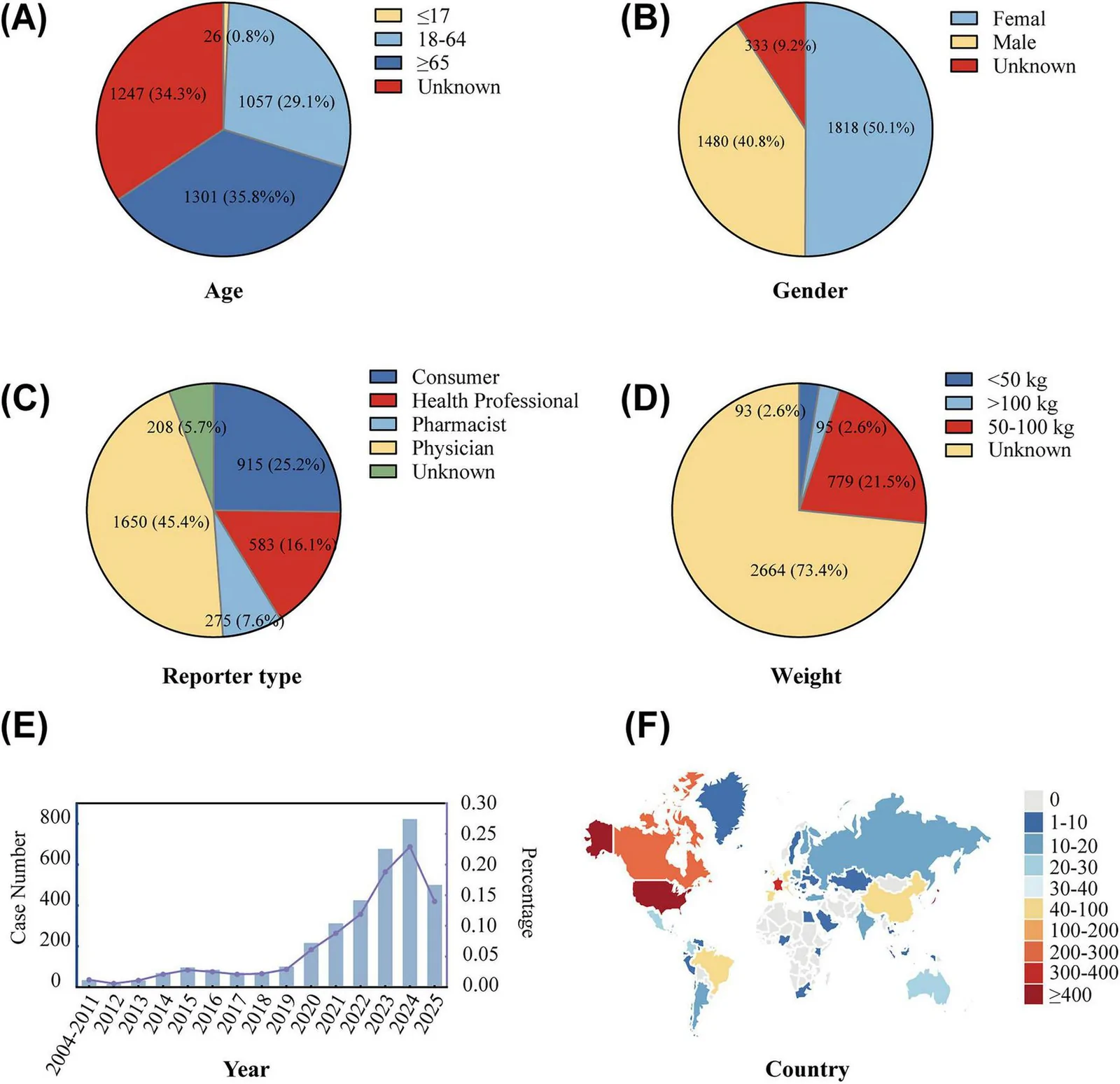
Demographic information on cutaneous toxicity with ADCs. **(A)** Age distribution of patients. **(B)** Gender distribution of patients. **(C)** Reporter types of CAE submissions. **(D)** Weight distribution of patients. **(E)** Annual number of ADC-related CAE reports in the FAERS (2004–2025). **(F)** Global geographical distribution of ADC-related CAE reports.

A total of 3,631 cases of CAEs associated with ADCs were identified from the FAERS database. The clinical characteristics of the included patients are summarized in Figure 2 (Supplementary Table 1). Demographic analysis revealed female patients accounted for 50.1% (*N* = 1,819) of all cases, while male patients represented 40.8% (*N* = 1,481). SG showed an overwhelming female predominance, followed by TV and T-DXd. In contrast, EV and BV were predominantly reported in male patients. The majority of patients were in the 65–85 years (33.6%) and 18–64 years (29.1%) age groups. Geographically, the top five countries with the most reports were the United States (40.8%), Japan (19.7%), France (8.8%), Canada (5.5%), and Germany (2.1%). Physicians submitted the highest proportion of reports (45.4%). Transitional cell carcinoma was the predominant indication (14.6%), followed by Hodgkin lymphoma (10.0%), bladder cancer (5.9%), and metastatic breast cancer (5.8%). Regarding report severity, GO had the highest proportion of serious reports (100%), followed by BV (87.8%) and EV (81.8%). In terms of clinical outcomes, hospitalization was reported in 23.4% of cases, followed by death (7.7%). Notably, GO was associated with the highest mortality rate (22.9%), whereas EV had the highest hospitalization rate (26.6%).

### 3.2 Categories of ADC-associated CAEs

The proportion of ADC-associated CAEs relative to the total AEs for each drug is illustrated in Supplementary Figures 1A (Supplementary Table 2). Notably, CAEs associated with EV accounted for 18.59% of all AEs documented for this ADC, while the corresponding proportions for Lonca, TV, and BV were markedly lower at 11.69, 8.04, and 4.12%. Further, Belamaf and GO showed the lowest CAE proportion, at 0.64 and 0.95%. Supplementary Figure 2 presents the distribution of CAEs at the HLT level. EV, Lonca, GO, and TV were mainly linked to the category of rashes, eruptions, and exanthems not elsewhere classified (NEC). Additionally, EV was associated with dermal and epidermal conditions (NEC), bullous conditions, pruritus (NEC), alopecia, dermatitis ascribed to specific agents, erythemas, and exfoliative conditions. The cutaneous toxicities of T-DM1 were predominantly telangiectasia and related conditions, nail and nail bed conditions (excluding infections and infestations), dermatitis ascribed to specific agents, and purpura and related conditions. T-DXd and SG were mainly associated with alopecia. BV was predominantly linked to dermatitis ascribed to specific agents, bullous conditions, dermatitis and eczema, the category of rashes, eruptions, and exanthems (NEC), and apocrine and eccrine gland disorders. Belamaf was primarily linked to photosensitivity and photodermatosis conditions. Supplementary Figure 1B presents a meta-style forest plot illustrating disproportionality across all treatment regimens. Results indicated that agent-specific risks varied substantially, with EV (ROR = 4.46), TV (ROR = 5.38), and Lonca (ROR = 2.75) exhibiting elevated risks.

### 3.3 Specific PTs on the cutaneous toxicity of ADCs

To further characterize the specific clinical manifestations at the PT level of the CAEs categorized above, we reconducted disproportionality signal analysis using ROR alongside the complementary IC method to assess these PT-level associations (Table 1 and Supplementary Table 3). Disproportionality analysis identified multiple robust, statistically significant signals. In the category of telangiectasia and related conditions, spider naevus showed an exceptionally prominent signal (ROR = 63.11, 95%CI = 39.25–101.46, *N* = 19), which was the highest among all CAE terms in this analysis. Among rashes, eruptions, and exanthems (NEC), vesicular rash, and maculo-papular rash exhibited strong associations (ROR = 2.78, 95%CI = 1.83–4.22, *N* = 22 and 2.39, 95%CI = 1.94–2.95, *N* = 88). For bullous conditions, the rare severe cutaneous event of SJS-TEN overlap exhibited a markedly stronger signal (ROR = 11.18, 95%CI = 5.98–20.92, *N* = 10). Strong associations were also identified for other rare CAEs: generalized exfoliative dermatitis (ROR = 5.98, 95%CI = 4.22–8.48, *N* = 32) in the “exfoliative conditions” category, and onycholysis (ROR = 4.33, 95%CI = 2.39–7.84, *N* = 11) in the “nail and nail bed conditions (excluding infections and infestations)” category. Notably, toxic erythema of chemotherapy (under the HLT of “dermatitis ascribed to specific agent”) and alopecia totalis (under the HLT of “alopecia”), two CAEs characterized by limited case numbers, exhibited elevated RORs: 44.97 (95%CI = 27.01–74.86, *N* = 16) and 5.17 (95%CI = 1.66–16.11, *N* = 3), respectively.

**TABLE 1:** Signal strength of ADC-related CAE at preferred terms (PT).

HLT	PT	Cases	ROR (95%Cl)	IC(95%CI)
Rashes, eruptions and exanthems nec	Rash	975	1.37 (1.29–1.46)	0.45 (0.36–0.54)
	Rash pruritic	94	1.05 (0.86–1.29)	0.08 (−0.22, −0.38)
	Rash maculo-papular	88	2.39 (1.94–2.95)	1.26 (0.93–1.59)
	Rash erythematous	82	1.11 (0.89–1.37)	0.14 (−0.18, −0.46)
	Rash macular	27	0.47 (0.32–0.68)	−1.10 (−1.62, −0.58)
	Rash vesicular	22	2.78 (1.83–4.22)	1.47 (0.76–2.18)
	Rash papular	19	0.49 (0.32–0.77)	−1.02 (−1.62, −0.42)
	Rash morbilliform	8	1.73 (0.87–3.47)	0.79 (−0.29, −1.87)
Alopecias	Alopecia	787	2.41 (2.24–2.58)	1.26 (1.15–1.37)
	Madarosis	11	0.53 (0.3–0.96)	–0.91 (−-1.68, –0.14)
	Alopecia totalis	3	5.17 (1.66–16.11)	2.36 (−-0.12, –4.84)
Erythemas	Erythema	255	0.74 (0.65–0.83)	–0.44 (−-0.62, –0.26)
	Palmar erythema	3	1.56 (0.5–4.85)	0.64 (−-0.99, –2.27)
Dermal and epidermal conditions nec	Skin disorder	172	3.11 (2.68–3.62)	1.63 (1.39–1.87)
	Dry skin	112	0.52 (0.43–0.62)	−0.95 (−1.21, −0.69)
	Skin toxicity	84	9.73 (7.84–12.08)	3.26 (2.80–3.72)
	Skin reaction	63	2.72 (2.12–3.48)	1.44 (1.04–1.84)
	Skin discolouration	59	0.76 (0.59–0.98)	−0.40 (−0.76, −0.04)
	Skin lesion	58	1.29 (1–1.67)	0.37 (−0.02, −0.76)
	Pain of skin	19	0.41 (0.26–0.64)	−1.28 (−1.88, −0.68)
	Skin burning sensation	17	0.14 (0.09–0.23)	−2.82 (−3.43, −2.21)
Bullous conditions	Stevens-johnson syndrome	145	3.84 (3.26–4.52)	1.93 (1.66–2.20)
	Toxic epidermal necrolysis	96	3.86 (3.16–4.72)	1.94 (1.61–2.27)
	Blister	90	0.98 (0.8–1.21)	−0.02 (−0.33, −0.29)
	Dermatitis bullous	56	4.65 (3.58–6.05)	2.21 (1.73–2.69)
	Erythema multiforme	25	1.59 (1.07–2.35)	0.67 (0.07–1.27)
	sjs-ten overlap	10	11.18 (5.98–20.92)	3.46 (1.64–5.28)
Urticarias	Urticaria	104	0.38 (0.31–0.46)	−1.39 (−1.67, −1.11)
Exfoliative conditions	Skin exfoliation	97	0.71 (0.58–0.86)	−0.50 (−0.79, −0.21)
	Dermatitis exfoliative generalized	32	5.98 (4.22–8.48)	2.57 (1.86–3.28)
	Exfoliative rash	13	2.94 (1.71–5.08)	1.55 (0.59–2.51)
Dermatitisascribed to specific agent	Drug eruption	60	2.08 (1.62–2.68)	1.06 (0.66–1.46)
	Palmar-plantar erythrodysaesthesia syndrome	43	1.08 (0.8–1.46)	0.11 (−0.33, −0.55)
	Drug reaction with eosinophilia and systemic symptoms	29	0.69 (0.48–1)	−0.53 (−1.04, −0.02)
	Toxic skin eruption	23	1.34 (0.89–2.02)	0.42 (−0.19, −1.03)
	Toxic erythema of chemotherapy	16	44.97 (27.01–74.86)	5.38 (2.89–7.87)
Apocrine and eccrine gland disorders	Hyperhidrosis	60	0.27 (0.21–0.35)	−1.87 (−2.22, −1.52)
	Night sweats	32	0.6 (0.43–0.85)	−0.73 (−1.22, −0.24)
Dermatitis and eczema	Dermatitis	43	1.29 (0.96–1.74)	0.37 (−0.08, −0.82)
	Eczema	33	0.46 (0.33–0.65)	−1.12 (−1.59, −0.65)
	Dermatitis allergic	20	1 (0.64–1.55)	0.00 (−0.63, −0.63)
	Skin irritation	14	0.18 (0.11–0.3)	−2.49 (−3.15, −1.83)
Nail and nail bed conditions (excl infections and infestations)	Onychoclasis	19	1.66 (1.06–2.61)	0.73 (0.04–1.42)
	Nail disorder	11	0.81 (0.45–1.46)	−0.31 (−1.12, −0.50)
	Onycholysis	11	4.33 (2.39–7.84)	2.11 (0.92–3.30)
Purpura and related conditions	Petechiae	23	1.33 (0.88–2)	0.41 (−0.20, −1.02)
	Purpura	12	0.8 (0.45–1.4)	−0.33 (−1.11, −0.45)
Skin and subcutaneous tissue ulcerations	Skin ulcer	27	0.62 (0.43–0.91)	−0.69 (−1.21, −0.17)
	Skin erosion	10	1.92 (1.03–3.57)	0.94 (−0.05, −1.93)
Acnes	Acne	16	0.12 (0.07–0.2)	−3.04 (−3.66, −2.42)
	Dermatitis acneiform	14	1.39 (0.82–2.35)	0.48 (−0.31, −1.27)
Telangiectasia and related conditions	Spider naevus	19	63.11 (39.25–101.46)	5.82 (3.22–8.42)
	Telangiectasia	17	10.93 (6.77–17.67)	3.42 (2.11–4.73)

**FIGURE 3:**
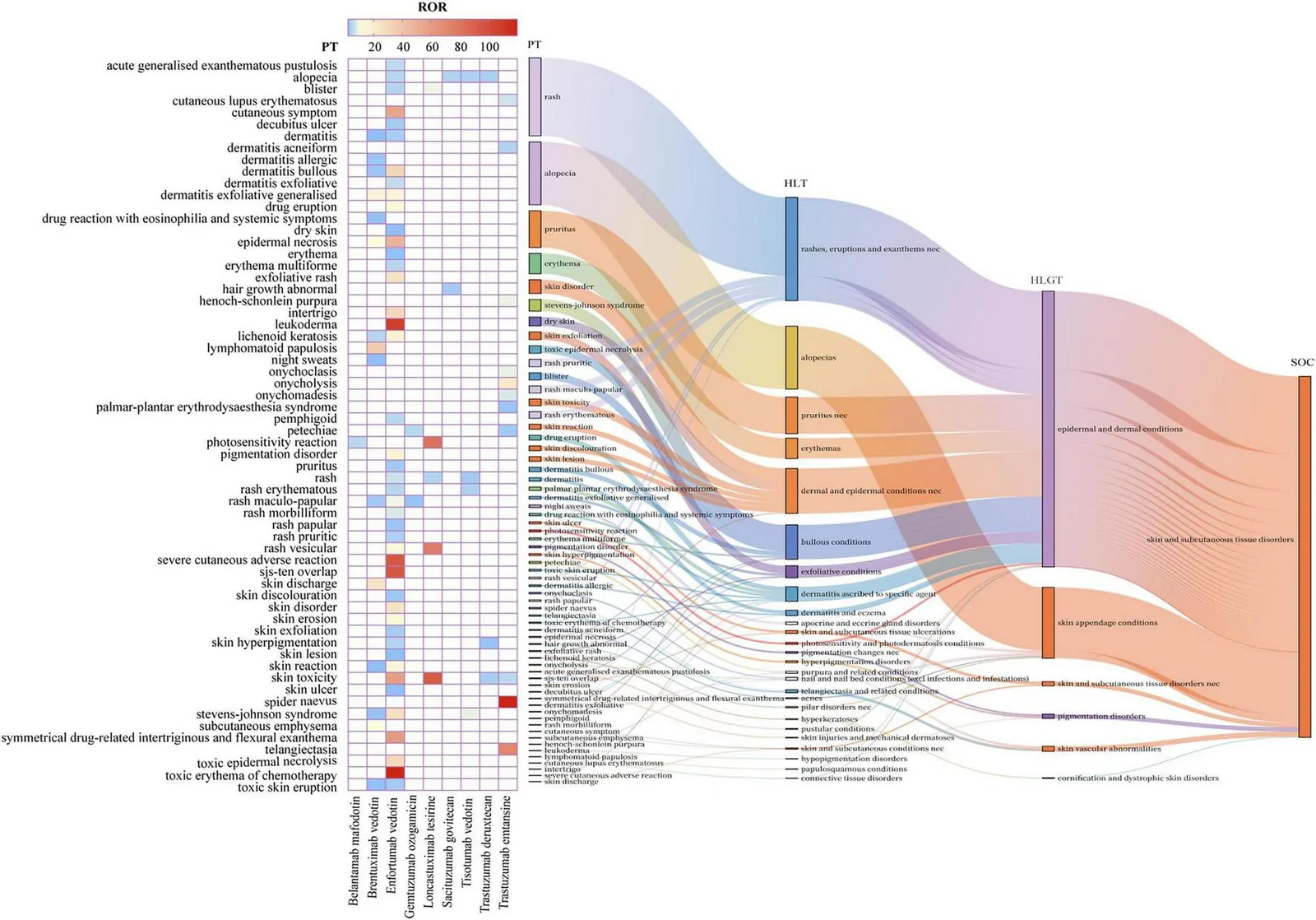
Scanning for ADC-related CAEs based on the FAERS database. The heatmap on the left shows the ROR for CAEs in the FAERS database under different ADC treatment strategies at the PT level. Sankey diagram on the right depicting the hierarchical relationship of PTs for ADC-related CAEs in MedDRA. PT indicates the preferred term, HLT indicates the high-level term, HLGT indicates the high-level group term, and SOC indicates the system organ class.

We conducted systematic visualization analyses of ADC-related CAEs by using the hierarchical classification framework of MedDRA. Specifically, the Sankey diagram depicted the hierarchical associations among these CAEs, which were sequentially categorized from SOC, through more specific HLGTs and HLTs, and ultimately to detailed PTs (Figure 3 and Supplementary Table 4). Concurrently, the heatmap in the left panel of Figure 3 displayed the RORs of distinct PTs across various ADC regimens (Supplementary Table 5). As observed from the number of colored cells in the heatmap, EV demonstrated the highest number of positive signals (44 positive signals), followed by BV (15 positive signals) and T-DM1 (11 positive signals). EV was strongly associated with specific PTs, including SJS-TEN overlap (ROR = 94.89), Symmetrical Drug-Related Intertriginous and Flexural Exanthema (SDRIFE; ROR = 59.44), and epidermal necrosis (ROR = 47.23). BV exhibited the strongest correlation with lymphomatoid papulosis (ROR = 36.15), while T-DM1 demonstrated a notable association with telangiectasia (ROR = 71.27) and spider naevus (ROR = 44.6). In contrast, Belamaf showed the weakest correlation with CAEs: among all PTs, only photosensitivity reaction had an ROR > 1 (ROR = 5.1).

### 3.4 Time-to-onset and outcome analysis of CAEs

**FIGURE 4:**
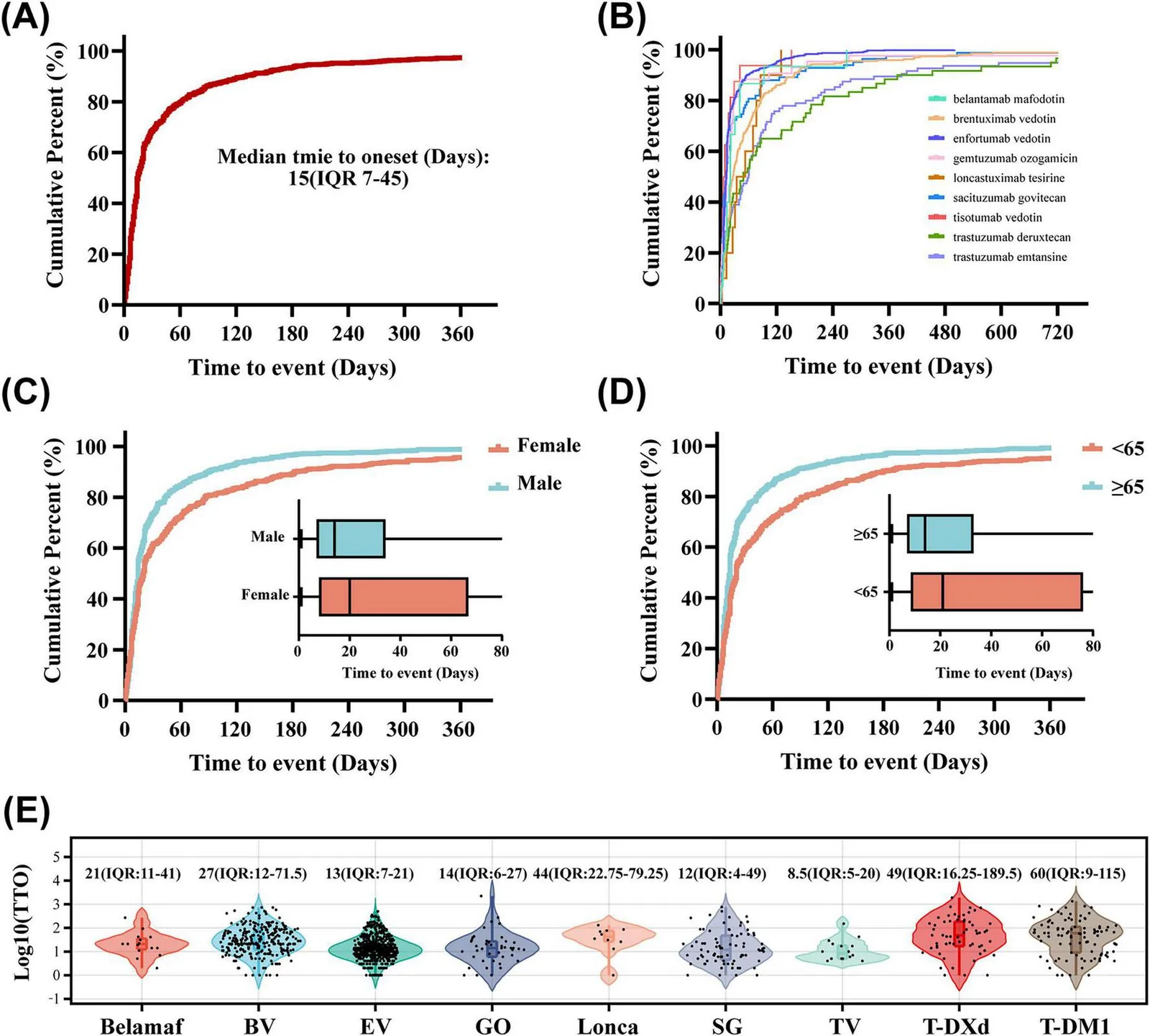
Time-to-onset analysis of ADC-related CAEs. **(A)** The cumulative distribution curves of the onset time of ADC-related CAEs. **(B)** The cumulative distribution curves of the onset time of ADC-related CAEs in different ADC treatment strategies. **(C)** The cumulative distribution curves of the onset time of ADC-related CAEs in gender subgroups. **(D)** The cumulative distribution curves of the onset time of ADC-related CAEs in age subgroups. **(E)** Distribution of log*10*-transformed time to onset (log*10*[TTO]) of CAEs associated with different ADCs.

Time-to-onset analysis was conducted on 1,192 CAE cases (32.83% of the total cohort) with complete time-to-onset data (Figure 4 and Supplementary Table 6). The median time to onset of CAEs was 15 days (interquartile range, IQR: 7–45). Notably, statistically significant differences in CAE onset time were observed across distinct ADC regimens: T-DXd exhibited the shortest median onset time (8.5 days, IQR: 5–20), whereas T-DM1 demonstrated the longest median onset time (60 days, IQR: 9–115). Subgroup analyses of CAE time to onset, stratified by gender and age, were further performed: the median onset time in male patients was 14 days (IQR: 7–34), which was earlier than that in female patients (20 days, IQR: 85–66.75). Patients aged < 65 years had a longer median onset time (21 days, IQR: 8.5–76) compared with those aged ≥ 65 years (14 days, IQR: 7–33). The Weibull distribution model further revealed that most ADCs conformed to an early failure pattern (shape parameter β < 1), indicative of a time-dependent reduction in CAE risk. In contrast, Lonca yielded a β estimate of 1.21 (95% CI: 0.58–1.84), a value numerically suggestive of a potential wear-out failure pattern; however, as its 95% CI includes 1, a definitive exclusion of either an early (β < 1) or random (β = 1) failure pattern is not statistically supported (Supplementary Table 7).

**FIGURE 5:**
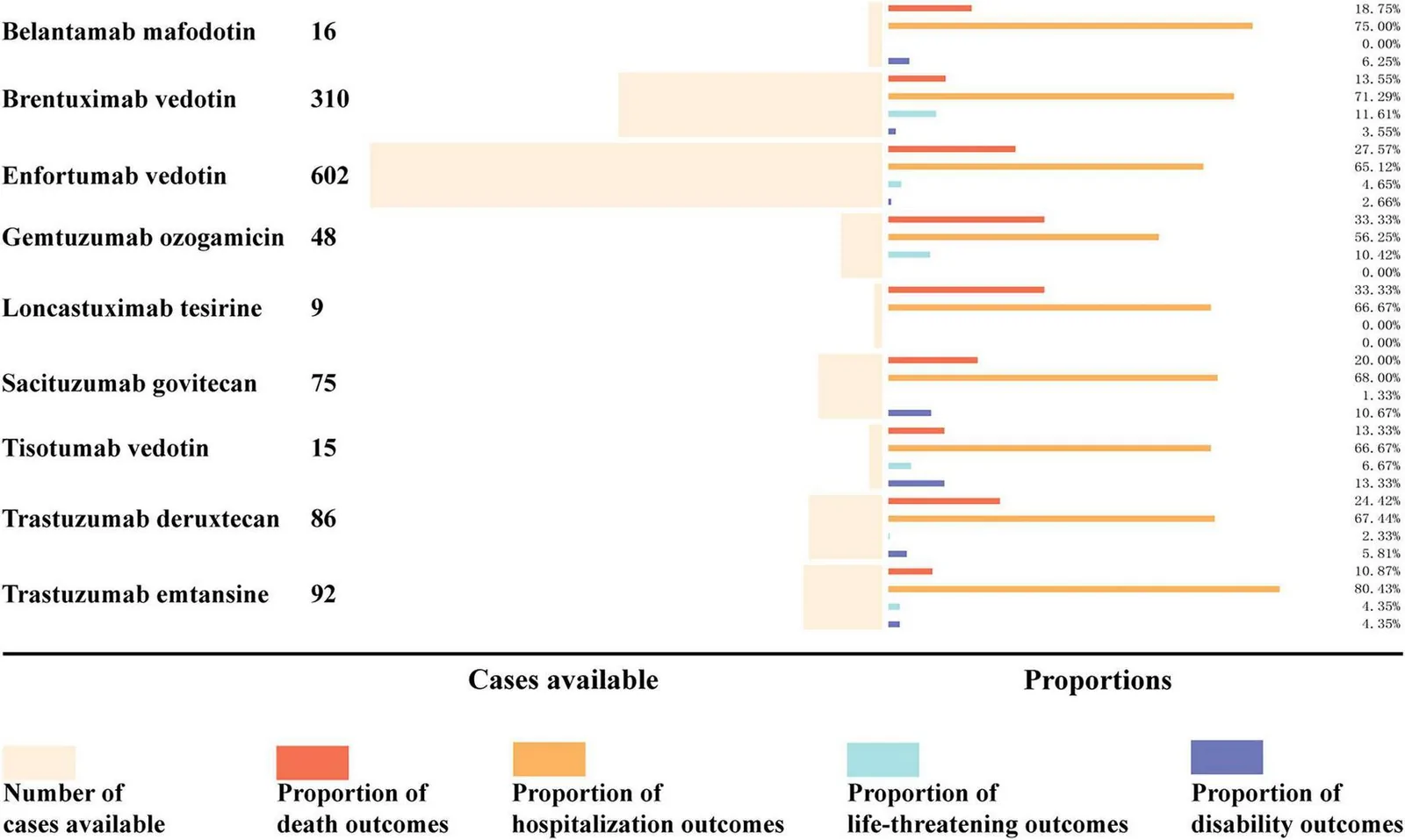
Outcome analysis of ADC-related CAEs. The number of cases, hospitalizations, and fatality proportions for ADC-associated CAEs were visualized.

To characterize the severity of clinical outcomes of ADC-associated CAEs, we analyzed the proportions of outcomes (including death, hospitalization, life-threatening events, and disability) across different ADC agents (Figure 5 and Supplementary Table 8). Hospitalization was the most common severe outcome of ADC-related CAEs: among all included drugs, T-DM1 had the highest hospitalization rate (80.43%), followed by Belamaf (75.00%) and BV (71.29%). Marked variations were also observed in mortality outcomes, with GO (33.33%) and the small-sample agent Lonca (33.33%) exhibiting the highest mortality proportions, whereas T-DM1 showed the lowest mortality rate (10.87%).

### 3.5 Head-to-head analysis of CAEs across different age and gender groups

To identify the key determinants of CAEs associated with ADCs, we utilized two-way stratified disproportionality analysis to conduct stratified analyses based on age and gender (Supplementary Figures 3, 4 and Supplementary Tables 9, 10). Results of the gender stratification showed that 27 ADC-related CAEs had significantly higher risks in male patients than in female patients, with the most notable risk disparities observed for toxic erythema of chemotherapy (ROR = 8.45, 95% CI: 2.41–29.65), acute generalized exanthematous pustulosis (ROR = 7.80, 95% CI: 1.66–36.72), and skin erosion (ROR = 7.80, 95% CI: 1.66–36.72); in contrast, only telangiectasia (ROR = 7.70, 95% CI: 1.02–58.28) and alopecia (ROR = 1.93, 95% CI: 1.61–2.31) exhibited significantly elevated risks in female patients. For the age stratification, elderly patients had significantly increased risks of multiple CAEs, including vesicular rash (ROR = 20.15, 95% CI: 2.65–153.68), SJS (ROR = 4.65, 95% CI: 2.88–77.51), whereas young patients only had higher risks of night sweats (ROR = 7.30, 95% CI: 1.71–31.14).

## 4 Discussion

ADCs demonstrate therapeutic advantages by minimizing off-target toxicities and enhancing antitumor efficacy. With the accumulation of compelling clinical efficacy evidence, ADCs have evolved from salvage therapy to a core standard-of-care strategy in oncology. Notably, several ADCs have achieved a pivotal transition from later-line therapies to first-line treatment (19, 20). However, the safety profile of ADCs with respect to CAEs, one of the most common adverse reactions associated with ADCs, remains insufficiently addressed. Although reports of ADC-related CAEs in randomized controlled trials (RCTs) have increased gradually, RCTs are inherently limited by strict inclusion criteria, relatively short follow-up durations, and protocol-driven monitoring strategies, and these limitations may lead to the underreporting or omission of delayed-onset or rare adverse events, particularly those with low incidence but severe clinical consequences (21). In contrast, real-world pharmacovigilance studies, which encompass more heterogeneous patient populations and capture longer-term safety data, are more likely to reveal toxicity patterns different from those observed in RCTs (22). Therefore, in the present study, we conducted a systematic evaluation of ADC-related CAEs using real-world case reports from the FAERS database in a post-marketing setting, aiming to provide direct evidence to inform clinical decision-making regarding ADC therapy. Our large-scale pharmacovigilance study, analyzing 3,631 CAEs identified from 104,195 AE reports, revealed substantial heterogeneity among individual agents. Furthermore, several severe and rare CAEs were found to exhibit strong regimen-specific associations. These findings confirm that CAEs pose a significant clinical challenge and hold substantial research value in ADC therapy. To our knowledge, this study has provided the earliest and most detailed characterization of ADC-related CAEs to date.

Descriptive analysis of the FAERS database showed a notable gender disparity in ADC-related CAEs. Approximately 85% of CAE reports for SG, TV, T-DXd, and T-DM1 were from female patients, whereas EV-related CAEs were predominantly reported in males (over 71.5%). This disparity is primarily driven by the sex-specific epidemiological profiles of their approved indications. Specifically, SG, T-DXd, and T-DM1 are mainly used for female-predominant breast cancer (23, 24)/TV targets female-exclusive cervical cancer (25), while EV is indicated for male-dominant urothelial carcinoma (26). To distinguish between inherent gender differences in CAE risk and this exposure-driven bias, Head-to-Head subgroup analysis revealed that male patients exhibited a higher relative risk of ADC-related CAEs than females did. The pattern is potentially attributed to the fact that higher androgen levels in males upregulate pro-inflammatory cytokines (e.g., IL-17, TNF-α) in keratinocytes via an androgen receptor-dependent pathway, thereby amplifying ADC-induced cutaneous inflammation (27, 28). Beyond gender, descriptive analysis revealed that the 65–85 years age group accounted for the highest reporting frequency of ADC-related CAEs, with stratified analysis further demonstrating an elevated relative reporting risk of multiple severe CAEs in elderly patients (≥ 65 years). However, we cannot exclude that this elevated risk is partially mediated by confounding factors highly prevalent in the elderly population, including greater comorbidity burden and polypharmacy. The observed association may also be driven by core biological pathways: age-related pharmacogenomic differences and cutaneous physiological decline (29). The cytotoxic payloads of ADCs are mainly metabolized by the cytochrome P450 enzyme family (30, 31), and reduced activity (20–40%) of these enzymes in the elderly (32) leads to payload accumulation in cutaneous tissues. Moreover, a progressive decrease in the number of Langerhans cells as age advances results in diminished capacity to regulate the ADC-associated inflammatory skin damage (33). Accordingly, advanced age should be considered a potential risk factor for ADC-related CAEs, with this association requiring further validation in prospective clinical studies with full adjustment for clinical confounders.

The time-to-onset analysis indicates that enfortumab vedotin has a shorter latency period for CAEs (median 13 days), which likely stems from its cleavable linker that facilitates the premature release of its highly cytotoxic payload (monomethyl auristatin E, MMAE). Conversely, T-DM1 has a longer latency (median 60 days), possibly due to the milder cytotoxicity of its payload (DM1) and the non-cleavable linker (34, 35). This underscores vigilance for potential late-onset CAEs in patients on prolonged therapy. Notably, our time-to-onset analysis has inherent limitations. First, using the documented ADC initiation date as the analysis time origin may introduce systematic bias due to incomplete or delayed documentation of post-discharge insidious-onset CAEs. Second, our analysis was restricted to 32.83% of the total cohort, potentially introducing selection bias and limiting the generalizability of findings. Geographically, most reports came from the U.S. and Japan, likely reflecting differences in drug approval, clinical adoption, and the maturity of the pharmacovigilance system, but not inherent biological susceptibility.

Our study demonstrated the value of a risk-stratified strategy for CAE prevention and management in patients treated with ADCs. At the SOC level, the results of our analysis demonstrated that three ADCs, namely EV, Lonca, and TV, were associated with a significantly higher proportion of CAEs among all AEs, and this association was statistically significant. At the PT level, the heatmap demonstrated that EV was linked to as many as 44 positive PTs, among which 6 signals reached the “extremely high-risk” level (ROR ≥ 40); notably, the RORs for SJS-TEN overlap syndrome, SDRIFE, and toxic erythema of chemotherapy were all extremely elevated. This finding was corroborated by clinical trial data: in the EV-301 and EV-302 studies, a considerable proportion of patients experienced treatment-related cutaneous reactions, with the incidence of grade 3–4 CAEs reaching approximately 15% (36, 37). Furthermore, post-marketing surveillance data have documented severe cutaneous adverse reactions linked to EV, including SJS, TEN, and SDRIFE (38, 39). These data mandate pre-treatment screening for a history of severe drug eruption and drug hypersensitivity to rigorously define EV treatment eligibility in high-risk populations, as well as continuous monitoring for hallmark warning signs of these fatal cutaneous toxicities throughout treatment, with immediate drug cessation and urgent dermatology referral upon suspicious presentations.

The expression of Nectin-4 in normal skin tissues is one of the key causes of severe cutaneous toxicity induced by EV (40); meanwhile, MMAE, the payload of EV, has been confirmed to exhibit a bystander killing effect, which can further exacerbate skin tissue damage (41). Additionally, we found that two other ADCs with MMAE as the payload, namely TV and BV, also exhibited cutaneous toxicities. TV showed a strong correlation with erythematous rash and SJS. Although BV did not show a significant correlation in the overall ROR analysis of cutaneous toxicities, 15 positive events were still detected at the PT level, specifically lymphomatoid papulosis and generalized exfoliative dermatitis. Therefore, given the shared bystander killing effect of the MMAE payload, lesion-specific and individualized cutaneous monitoring should be routinely implemented for all patients treated with MMAE-conjugated ADCs throughout treatment. For Lonca, we identified a strong association with photosensitivity reactions and blisters, which is also supported by clinical trials and case reports (42, 43). Mechanistically, it is hypothesized that the pyrrolobenzodiazepine (PBD) payload of Lonca induces off-target cutaneous DNA toxicity, which increases the sensitivity of skin cells to ultraviolet radiation, leading to cutaneous toxicities that mostly occur in sun-exposed areas. Secondly, the bystander effect may exacerbate the progression of cutaneous toxicities by affecting CD19-negative cells (44, 45). Clinically, this mandates pre-treatment photosensitivity education and strict sun protection guidance for Lonca-treated patients, with continuous monitoring for erythema and blistering in sun-exposed areas throughout therapy.

Furthermore, we identified several highly relevant CAEs associated with other ADCs: T-DM1 was strongly linked to spider naevus and telangiectasia. This may be attributed to the fact that emtansine (DM1) disrupts cytoskeletal microtubules and induces vascular dilation, which collectively contribute to the development of telangiectasia (46, 47). Notably, CAEs are not listed in the prescribing information (PI) for Belamaf (48), whereas only alopecia, rash, pruritus, and dry skin are documented in the PIs for GO, SG, and T-DXd (49–51). However, our findings demonstrated that these ADCs were associated with a low risk of additional cutaneous toxicities. Specifically, Belamaf may induce photosensitivity reactions, while GO was strongly correlated with maculopapular rash and petechiae. Furthermore, beyond the previously reported alopecia, SG was associated with abnormal hair growth, and T-DXd was linked to skin hyperpigmentation. Healthcare providers should be cognizant of these potential cutaneous toxicities to enable timely detection and management of cutaneous complications.

Collectively, the mechanistic insights derived from our study underscore that ADC-induced cutaneous toxicity is a multifactorial process. With respect to payload-dependent toxicity, our data corroborate that distinct cytotoxic moieties (i.e., MMAE, PBD, DM1) elicit divergent cutaneous toxicity phenotypes. While our study does not provide direct experimental or histopathological evidence for the underlying molecular pathways, we hypothesize that these phenotypic differences may be mediated by payload-specific mechanisms. Additionally, the expression of target antigens in normal cutaneous tissues exacerbates on-target toxicity. This mechanistic framework also explains why the paucity of prominent cutaneous toxicity signals associated with GO can be partially ascribed to the restricted expression of its target antigen (CD33) in skin tissues (52).

Currently, the clinical management of ADC-related CAEs is centered on inflammatory control and local symptom alleviation. For mild grade 1 CAEs, local symptomatic treatment is preferred, typically involving topical steroids, emollients, or oral antihistamines. Grade 2 CAEs require systemic corticosteroids, along with ADC dose adjustment or temporary treatment hold based on symptom severity. Severe grade ≥ 3 cutaneous toxicities (e.g., SJS, TEN, generalized exfoliative dermatitis), which may induce multi-organ damage or even be life-threatening, warrant permanent discontinuation of treatment (44, 53–55). Accordingly, pre-treatment baseline skin evaluation (particularly in susceptible patient populations), close surveillance of cutaneous manifestations during the high-risk clinical window, and prompt skin biopsy for atypical or rapidly progressive CAEs represent pivotal strategies to prevent the escalation of mild CAEs to severe forms. These interventions ultimately safeguard treatment safety while optimizing the antitumor efficacy of ADCs in clinical practice.

Nevertheless, this study has several limitations. First, the core methodological limitation of the FAERS database is its lack of no true exposure denominator, precluding calculation of the true incidence of ADC-related CAEs. All findings are derived from pharmacovigilance signals (ROR, IC), which only reflect relative reporting associations. Second, FAERS has inherent limitations including underreporting, selection bias, variable data quality, and inability to fully exclude confounding from underlying comorbidities and concomitant medications. Furthermore, the lack of key clinical details (e.g., dosage, complications) restricts comprehensive risk assessment and long-term outcome evaluation. Finally, variations in reporting standards and procedures across different national healthcare systems lead to inconsistencies in data completeness and quality. Despite these inherent limitations, the FAERS database remains an invaluable tool for identifying safety signals in large patient populations. By leveraging large-scale data and rigorous analytical strategies, this study comprehensively delineated the potential risks and signal spectrum of ADC-induced CAEs. The elucidation of underlying mechanisms will deepen the understanding of ADC-related cutaneous toxicity. These insights lay a foundation for future research and offer meaningful guidance to improve the safety, tolerability, and overall clinical management of ADC therapies.

## 5 Conclusion

By utilizing data mining methods, this study provides a comprehensive analysis of CAEs associated with ADCs. Our findings reveal substantial heterogeneity in CAEs among different ADCs, identify several novel and severe regimen-specific CAEs, and clarify the multifactorial mechanisms underlying ADC-induced cutaneous toxicity, which may be associated with the type of cytotoxic payload and the expression of targets in skin tissues. In summary, these findings can enhance clinicians’ awareness of potential ADC-associated CAEs, and particularly facilitate the establishment of risk alertness in the early phase of ADC treatment; timely identification and proactive intervention based on this awareness are critical for mitigating or preventing the severe clinical consequences induced by such adverse reactions.

## Data Availability

The original contributions presented in the study are included in the article/Supplementary material, further inquiries can be directed to the corresponding author.

## References

[1] Tarantino P Carmagnani Pestana R Corti C Modi S Bardia A Tolaney SM et al. Antibody-drug conjugates: smart chemotherapy delivery across tumor histologies. *CA: Cancer J Clin.* (2022) 72:165–82. 10.3322/caac.21705 34767258

[2] Tolcher AW. Antibody drug conjugates: lessons from 20 years of clinical experience. *Ann Oncol.* (2016) 27:2168–72. 10.1093/annonc/mdw424 27733376

[3] Khongorzul P Ling CJ Khan FU Ihsan AU Zhang J. Antibody-Drug conjugates: a comprehensive review. *Mol. Cancer Res MCR.* (2020) 18:3–19. 31659006 10.1158/1541-7786.MCR-19-0582

[4] Shitara K Van Cutsem E Gümüş M Lonardi S de la Fouchardière C Coutzac C et al. Trastuzumab deruxtecan or ramucirumab plus paclitaxel in gastric Cancer. *New Engl J Med.* (2025) 393:336–48. 10.1056/NEJMoa2503119 40454632

[5] Cortés J Punie K Barrios C Hurvitz SA Schneeweiss A Sohn J et al. Sacituzumab govitecan in untreated, advanced triple-negative Breast Cancer. *New Engl J Med.* (2025) 393:1912–25. 10.1056/NEJMoa2511734 41124233

[6] Beck A Goetsch L Dumontet C Corvaïa N. Strategies and challenges for the next generation of antibody-drug conjugates. *Nat Rev Drug Discovery.* (2017) 16:315–37. 10.1038/nrd.2016.268 28303026

[7] Chen B Zheng X Wu J Chen G Yu J Xu Y et al. Antibody-drug conjugates in cancer therapy: current landscape, challenges, and future directions. *Mol Cancer.* (2025) 24:279. 10.1186/s12943-025-02489-2 41184856 PMC12581584

[8] Tan HN Morcillo MA Lopez J Minchom A Sharp A Paschalis A et al. Treatment-related adverse events of antibody drug-conjugates in clinical trials. *J Hematol Oncol.* (2025) 18:71. 10.1186/s13045-025-01720-3 40611310 PMC12231679

[9] Gronbeck C Hadfield MJ Grant-Kels JM. Dermatologic toxicities of antibody-drug conjugates. *J Am Acad Dermatol.* (2024) 91:1177–88. 10.1016/j.jaad.2024.08.036 39182677

[10] Zhu Y Liu K Wang K Zhu H. Treatment-related adverse events of antibody-drug conjugates in clinical trials: a systematic review and meta-analysis. *Cancer.* (2023) 129:283–95. 10.1002/cncr.34507 36408673 PMC10099922

[11] Powles T Rosenberg JE Sonpavde GP Loriot Y Durán I Lee JL et al. Enfortumab vedotin in previously treated advanced urothelial carcinoma. *New Engl J Med.* (2021) 384:1125–35. 10.1056/NEJMoa2035807 33577729 PMC8450892

[12] Hu X Curigliano G Yonemori K Bardia A Barrios CH Sohn J et al. Patient-reported outcomes with trastuzumab deruxtecan in hormone receptor-positive, HER2-low or HER2-ultralow metastatic breast cancer: results from the randomized DESTINY-Breast06 trial. *ESMO Open.* (2025) 10:105082. 10.1016/j.esmoop.2025.105082 40441802 PMC12167881

[13] Tarantino P Ricciuti B Pradhan SM Tolaney SM. Optimizing the safety of antibody-drug conjugates for patients with solid tumours. *Nat Rev. Clin Oncol.* (2023) 20:558–76. 10.1038/s41571-023-00783-w 37296177

[14] Chang HP Le HK Shah DK. Pharmacokinetics and pharmacodynamics of antibody-drug conjugates administered via subcutaneous and intratumoral routes. *Pharmaceutics.* (2023) 15:1132. 10.3390/pharmaceutics15041132 37111619 PMC10142912

[15] Sakaeda T Tamon A Kadoyama K Okuno Y. Data mining of the public version of the FDA adverse event reporting system. *Intern J Med Sci.* (2013) 10:796–803. 10.7150/ijms.6048 23794943 PMC3689877

[16] Bate A Lindquist M Edwards IR Olsson S Orre R Lansner A et al. A Bayesian neural network method for adverse drug reaction signal generation. *Eur J Clin Pharmacol.* (1998) 54:315–21. 10.1007/s002280050466 9696956

[17] Cui Z Cheng F Wang L Zou F Pan R Tian Y et al. A pharmacovigilance study of etoposide in the FDA adverse event reporting system (FAERS) database, what does the real world say? *Front Pharmacol.* (2023) 14:1259908. 10.3389/fphar.2023.1259908 37954852 PMC10637489

[18] Tang Y She Y Chen D Zhou Y Liu Z Chen Z et al. Signal mining and analysis of influencing factors for adverse events of Nivolumab and Cetuximab in the treatment of head and neck cancer based on the US FAERS database. *Front Immunol.* (2025) 16:1658535. 10.3389/fimmu.2025.1658535 41235256 PMC12605481

[19] National Comprehensive Cancer Network *NCCN Clinical Practice Guidelines in Oncology: Breast Cancer [Guideline].* Plymouth Meeting, PA: NCCN (2026).

[20] National Comprehensive Cancer Network *NCCN Clinical Practice Guidelines in Oncology: Bladder Cancer [Guideline].* Plymouth Meeting, PA: NCCN (2025).

[21] Othus M Freidlin B Korn EL. Avoiding delays in reporting time-to-event randomized trials: calendar backstops and other approaches. *J Clin Oncol.* (2024) 42:3753–60. 10.1200/jco.24.00025 38759123 PMC11521763

[22] Song S Yang Y Hu Q Zhong R Lei X Wang C et al. Infectious adverse events associated with immune checkpoint inhibitors: a pharmacovigilance analysis based on FAERS database. *Front Immunol.* (2025) 16:1647944. 10.3389/fimmu.2025.1647944 41208964 PMC12589035

[23] Swain SM Shastry M Hamilton E. Targeting HER2-positive breast cancer: advances and future directions. *Nat Rev Drug Discovery.* (2023) 22:101–26. 10.1038/s41573-022-00579-0 36344672 PMC9640784

[24] Xu Y Gong M Wang Y Yang Y Liu S Zeng Q. Global trends and forecasts of breast cancer incidence and deaths. *Sci Data.* (2023) 10:334. 10.1038/s41597-023-02253-5 37244901 PMC10224917

[25] Markham A. Tisotumab vedotin: first approval. *Drugs.* (2021) 81:2141–7. 10.1007/s40265-021-01633-8 34748188

[26] Moussa M Papatsoris A Abou Chakra M Dellis A. Profile of enfortumab vedotin in the treatment of urothelial carcinoma: the evidence to date. *Drug Design Dev Therapy.* (2021) 15:453–62. 10.2147/dddt.S240854 33603337 PMC7886109

[27] Gilliver SC Wu F Ashcroft GS. Regulatory roles of androgens in cutaneous wound healing. *Thrombosis Haemostasis.* (2003) 90:978–85. 10.1160/th03-05-0302 14652627

[28] Lai JJ Lai KP Chuang KH Chang P Yu IC Lin WJ et al. Monocyte/macrophage androgen receptor suppresses cutaneous wound healing in mice by enhancing local TNF-alpha expression. *J Clin Invest.* (2009) 119:3739–51. 10.1172/jci39335 19907077 PMC2786793

[29] Skossyrskiy V Boot M Gadaev I Sekacheva M Orlova E. Drug-induced cutaneous toxicities in solid tumor oncology: mechanisms, manifestations, and management. *Med Oncol.* (2025) 43:52. 10.1007/s12032-025-03193-3 41381986

[30] Han TH Zhao B. Absorption, distribution, metabolism, and excretion considerations for the development of antibody-drug conjugates. *Drug Metab Dispos.* (2014) 42:1914–20. 10.1124/dmd.114.058586 25048520

[31] Chang HP Cheung YK Shah DK. Whole-Body pharmacokinetics and physiologically based pharmacokinetic Model for Monomethyl Auristatin E (MMAE). *J Clin Med.* (2021) 10:1332. 10.3390/jcm10061332 33807057 PMC8004929

[32] Sotaniemi EA Arranto AJ Pelkonen O Pasanen M. Age and cytochrome P450-linked drug metabolism in humans: an analysis of 226 subjects with equal histopathologic conditions. *Clin Pharmacol Therapeut.* (1997) 61:331–9. 10.1016/s0009-9236(97)90166-1 9091249

[33] Pilkington SM Dearman RJ Kimber I Griffiths CEM. Langerhans cells express human β-defensin 3: relevance for immunity during skin ageing. *Br J Dermatol.* (2018) 179:1170–1. 10.1111/bjd.16770 29758092

[34] Tang H Liu Y Yu Z Sun M Lin L Liu W et al. The analysis of key factors related to ADCs structural design. *Front Pharmacol.* (2019) 10:373. 10.3389/fphar.2019.00373 31068807 PMC6491742

[35] Jin Y Schladetsch MA Huang X Balunas MJ Wiemer AJ. Stepping forward in antibody-drug conjugate development. *Pharmacol Therapeut.* (2022) 229:107917. 10.1016/j.pharmthera.2021.107917 34171334 PMC8702582

[36] Powles T Valderrama BP Gupta S Bedke J Kikuchi E Hoffman-Censits J et al. Enfortumab vedotin and pembrolizumab in untreated advanced urothelial Cancer. *New Engl J Med.* (2024) 390:875–88. 10.1056/NEJMoa2312117 38446675

[37] Rosenberg JE Powles T Sonpavde GP Loriot Y Duran I Lee JL et al. EV-301 long-term outcomes: 24-month findings from the phase III trial of enfortumab vedotin versus chemotherapy in patients with previously treated advanced urothelial carcinoma. *Ann Oncol.* (2023) 34:1047–54. 10.1016/j.annonc.2023.08.016 37678672

[38] Vlachou E Matoso A McConkey D Jing Y Johnson BA Hahn NM et al. Enfortumab vedotin-related cutaneous toxicity and radiographic response in patients with urothelial Cancer: a single-center experience and review of the literature. *Eur Urol Open Sci.* (2023) 49:100–3. 10.1016/j.euros.2023.01.002 36820243 PMC9937876

[39] Nguyen MN Reyes M Jones SC. Postmarketing cases of enfortumab vedotin-associated skin reactions reported as stevens-johnson syndrome or toxic epidermal necrolysis. *JAMA Dermatol.* (2021) 157:1237–9. 10.1001/jamadermatol.2021.3450 34495281 PMC8427493

[40] Fortugno P Josselin E Tsiakas K Agolini E Cestra G Teson M et al. Nectin-4 mutations causing ectodermal dysplasia with syndactyly perturb the rac1 pathway and the kinetics of adherens junction formation. *J Invest Dermatol.* (2014) 134:2146–53. 10.1038/jid.2014.119 24577405

[41] Saberi SA Cheng D Nambudiri VE. Antibody-drug conjugates: a review of cutaneous adverse effects. *J Am Acad Dermatol.* (2024) 91:922–31. 10.1016/j.jaad.2024.07.1463 39047980

[42] Gociman S Baron K Hu B Zussman J Madigan LM. Blistering lesions associated with loncastuximab tesirine. *JAMA Dermatol.* (2022) 158:831–2. 10.1001/jamadermatol.2022.1389 35583892

[43] Epperla N Olszewski AJ Ayers EC Ahmed S. Management of adverse reactions to loncastuximab in patients with relapsed or refractory diffuse large B-cell lymphoma. *Hematol Oncol.* (2025) 43:e70128. 10.1002/hon.70128 40836271 PMC12368254

[44] Sorensen EP Thrush J Bartlett NL Rosman IS Anadkat MJ Jones HA. Diffuse telangiectatic rash associated with novel antibody drug conjugate therapies. *JAMA Dermatol.* (2020) 156:601–3. 10.1001/jamadermatol.2020.0208 32267477

[45] Juárez-Salcedo LM Nimkar S Corazón AM Dalia S. Loncastuximab tesirine in the treatment of relapsed or refractory diffuse large B-cell lymphoma. *Intern J Mol Sci.* (2024) 25:7580. 10.3390/ijms25147580 39062823 PMC11276998

[46] Sibaud V Vigarios E Combemale P Lamant L Lacouture ME Lacaze JL et al. T-DM1-related telangiectasias: a potential role in secondary bleeding events. *Ann Oncol.* (2015) 26:436–7. 10.1093/annonc/mdu533 25403586

[47] Kwon Y Gomberg-Maitland M Pritzker M Thenappan T. Telangiectasia and pulmonary arterial hypertension following treatment with trastuzumab emtansine: a case report. *Chest.* (2016) 149:e103–5. 10.1016/j.chest.2015.09.008 27055712

[48] U.S. Food and Drug Administration *BLENREP (belantamab mafodotin-blmf) for Injection, for Intravenous Use [Prescribing Information].* Silver Spring, MD: U.S. Food and Drug Administration (2025).

[49] U.S. Food and Drug Administration *FASENRA (benralizumab) Injection, for Subcutaneous Use [Prescribing Information].* Silver Spring, MD: U.S. Food and Drug Administration (2017).

[50] U.S. Food and Drug Administration *TAKHZYRO (lanadelumab-flyo) injection, for Subcutaneous Use [Prescribing Information].* Silver Spring, MD: U.S. Food and Drug Administration (2020).

[51] U.S. Food and Drug Administration *POLIVY (polatuzumab vedotin-piiq) for Injection, for Intravenous Use [Prescribing Information].* Silver Spring, MD: U.S. Food and Drug Administration (2019).

[52] McGavin JK Spencer CM. Gemtuzumab ozogamicin. *Drugs.* (2001) 61:1317–22; discussion 23–4. 10.2165/00003495-200161090-00007. 11511025

[53] Lacouture ME Patel AB Rosenberg JE O’Donnell PH. Management of dermatologic events associated with the Nectin-4-directed antibody-drug conjugate enfortumab vedotin. *Oncologist.* (2022) 27:e223–32. 10.1093/oncolo/oyac001 35274723 PMC8914492

[54] Ingen-Housz-Oro S Elshot YS Segura S Marchand A Pouessel D Kluger N et al. Skin toxicity of enfortumab vedotin: proposal of a specific management algorithm. *J Eur Acad Dermatol Venereol JEADV.* (2024) 38:e99–101. 10.1111/jdv.19454 37607297

[55] Spring LM Nakajima E Hutchinson J Viscosi E Blouin G Weekes C et al. Sacituzumab govitecan for metastatic triple-negative breast cancer: clinical overview and management of potential toxicities. *Oncologist.* (2021) 26:827–34. 10.1002/onco.13878 34176192 PMC8488774

