# Supplementary material for: Cutaneous adverse events with antibody-drug conjugates: a FAERS-based pharmacovigilance study
Source: Front Med (Lausanne). 2026 May 25;13:1847032. doi: 10.3389/fmed.2026.1847032 (PMC13243105; doi:10.3389/fmed.2026.1847032)
Supplement: Supplementary file 2 [file Data_Sheet_1.pdf]

(A)

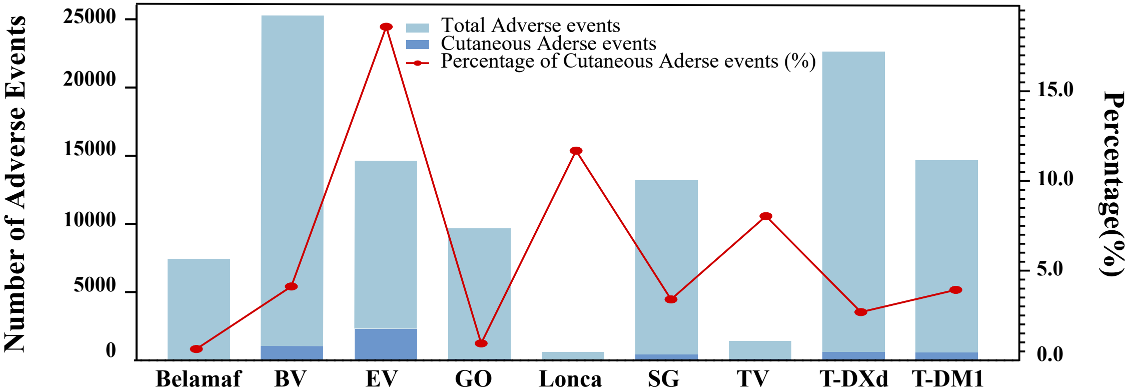

(B)

Drug name	Cases	ROR	95%Cl
belantamab mafodotin	47	0.24	0.18-0.32
brentuximab vedotin	998	0.86	0.81-0.92
enfortumab vedotin	2288	4.46	4.26-4.66
gemtuzumab ozogamicin	91	0.32	0.26-0.39
loncastuximab tesirine	61	5.38	4.12-7.03
sacituzumab govitecan	433	0.81	0.73-0.89
tisotumab vedotin	104	2.75	2.25-3.36
trastuzumab deruxtecan	592	0.59	0.55-0.64
trastuzumab emtansine	555	0.88	0.81-0.96

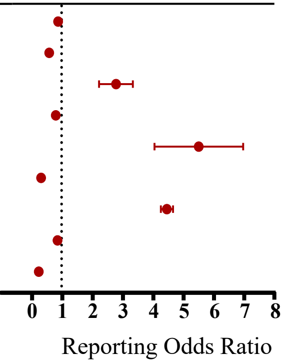

Supplementary Figure 1. (A) Proportion of cutaneous adverse events relative to total adverse events for each ADC. (B) Forest plot of ROR and 95% CI for CAEs associated with each ADC.
