# Supplementary material for: Cutaneous adverse events with antibody-drug conjugates: a FAERS-based pharmacovigilance study
Source: Front Med (Lausanne). 2026 May 25;13:1847032. doi: 10.3389/fmed.2026.1847032 (PMC13243105; doi:10.3389/fmed.2026.1847032)
Supplement: Supplementary file 3 [file Data_Sheet_2.pdf]

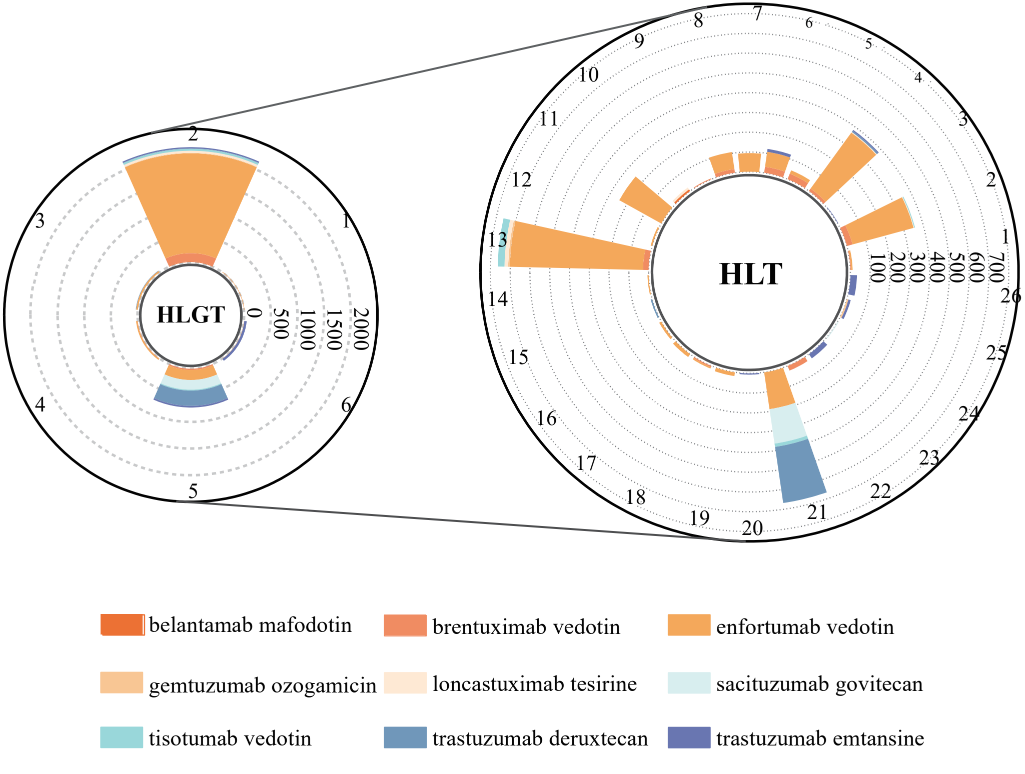

Supplementary Figure 2. Distribution of Preferred Terms (PTs) for CAEs across 9 ADCs.
HLGT circle: 1=cornification and dystrophic skin disorders; 2=epidermal and dermal conditions; 3=pigmentation disorders; 4=skin and subcutaneous tissue disorders nec; 5=skin appendage conditions; 6=skin vascular abnormalities;
HLT circle: 1=acnes, 2=alopecias, 3=apocrine and eccrine gland disorders, 4=bullous conditions, 5=connective tissue disorders, 6=dermal and epidermal conditions nec, 7=dermatitis and eczema, 8=dermatitis ascribed to specific agent, 9=erythemas, 10=exfoliative conditions, 11=hyperkeratosis, 12=hyperpigmentation disorders, 13=hypopigmentation disorders, 14=nail and nail bed conditions (excl infections and infestations), 15=papulosquamous conditions, 16=photosensitivity and photodermatosis conditions, 17=pigmentation changes nec, 18=pilar disorders nec, 19=pruritus nec, 20=purpura and related conditions, 21=pustular conditions, 22=rashes, eruptions and exanthems nec, 23=skin and subcutaneous conditions nec, 24=skin and subcutaneous tissue ulcerations, 25=skin injuries and mechanical dermatoses, 26=telangiectasia and related conditions.
