# Supplementary material for: Cutaneous adverse events with antibody-drug conjugates: a FAERS-based pharmacovigilance study
Source: Front Med (Lausanne). 2026 May 25;13:1847032. doi: 10.3389/fmed.2026.1847032 (PMC13243105; doi:10.3389/fmed.2026.1847032)
Supplement: Supplementary file 4 [file Data_Sheet_3.pdf]

PT	Female/Male	ROR(95%CI)
acute generalised exanthematous pustulosis	2/8	0.13 (0.03 - 0.6)
alopecia	566/151	1.93 (1.61 - 2.31)
blister	48/41	0.6 (0.4 - 0.91)
cutaneous lupus erythematosus	3/0	\
cutaneous symptom	1/6	0.09 (0.01 - 0.71)
decubitus ulcer	3/6	0.26 (0.06 - 1.03)
dermatitis	23/17	0.69 (0.37 - 1.3)
dermatitis acneiform	11/0	\
dermatitis allergic	7/12	0.3 (0.12 - 0.76)
dermatitis bullous	15/39	0.2 (0.11 - 0.36)
dermatitis exfoliative	4/4	0.51 (0.13 - 2.05)
dermatitis exfoliative generalised	9/16	0.29 (0.13 - 0.65)
drug eruption	19/38	0.26 (0.15 - 0.44)
drug reaction with eosinophilia and systemic symptoms	16/12	0.68 (0.32 - 1.45)
dry skin	56/48	0.6 (0.41 - 0.88)
epidermal necrosis	4/10	0.21 (0.06 - 0.65)
erythema	139/103	0.69 (0.54 - 0.89)
erythema multiforme	13/9	0.74 (0.32 - 1.73)
exfoliative rash	4/8	0.26 (0.08 - 0.85)
hair growth abnormal	12/0	\
henoch -schonlein purpura	4/0	\
intertrigo	0/3	\
leukoderma	1/3	0.17 (0.02 - 1.64)
lichenoid keratosis	3/8	0.19 (0.05 - 0.73)
lymphomatoid papulosis	1/3	0.17 (0.02 - 1.64)
night sweats	14/12	0.6 (0.28 - 1.29)
onychoclasis	11/0	\
onycholysis	7/2	1.8 (0.37 - 8.65)
onychomadesis	5/1	2.57 (0.3 - 21.96)
palmar -plantar erythrodysaesthesia syndrome	28/11	1.31 (0.65 - 2.62)
pemphigoid	2/6	0.17 (0.03 - 0.85)
petechiae	13/10	0.67 (0.29 - 1.52)
photosensitivity reaction	8/7	0.59 (0.21 - 1.62)
pigmentation disorder	10/12	0.43 (0.18 - 0.99)
pruritus	216/208	0.53 (0.44 - 0.64)
rash	418/464	0.46 (0.4 - 0.52)
rash erythematous	31/44	0.36 (0.23 - 0.57)
rash maculo -papular	37/43	0.44 (0.28 - 0.68)
rash morbilliform	3/5	0.31 (0.07 - 1.29)
rash papular	7/12	0.3 (0.12 - 0.76)
rash pruritic	51/39	0.67 (0.44 - 1.02)
rash vesicular	11/11	0.51 (0.22 - 1.18)
severe cutaneous adverse reaction	1/2	0.26 (0.02 - 2.83)
sjs-ten overlap	3/6	0.26 (0.06 - 1.03)
skin discharge	0/3	\
skin discolouration	33/21	0.81 (0.47 - 1.39)
skin disorder	54/109	0.25 (0.18 - 0.35)
skin erosion	2/8	0.13 (0.03 - 0.6)
skin exfoliation	40/48	0.43 (0.28 - 0.65)
skin hyperpigmentation	15/8	0.96 (0.41 - 2.27)
skin lesion	26/27	0.49 (0.29 - 0.85)
skin reaction	24/37	0.33 (0.2 - 0.56)
skin toxicity	28/51	0.28 (0.18 - 0.45)
skin ulcer	13/12	0.56 (0.25 - 1.22)
spider naevus	18/0	\
stevens -johnson syndrome	46/88	0.27 (0.19 - 0.38)
subcutaneous emphysema	5/1	2.57 (0.3 - 21.96)
symmetrical drug-related intertriginous and flexural exanthema	0/9	\
telangiectasia	15/1	7.7 (1.02 - 58.28)
toxic epidermal necrolysis	24/67	0.18 (0.12 - 0.29)
toxic erythema of chemotherapy	3/13	0.12 (0.03 - 0.42)
toxic skin eruption	13/9	0.74 (0.32 - 1.73)

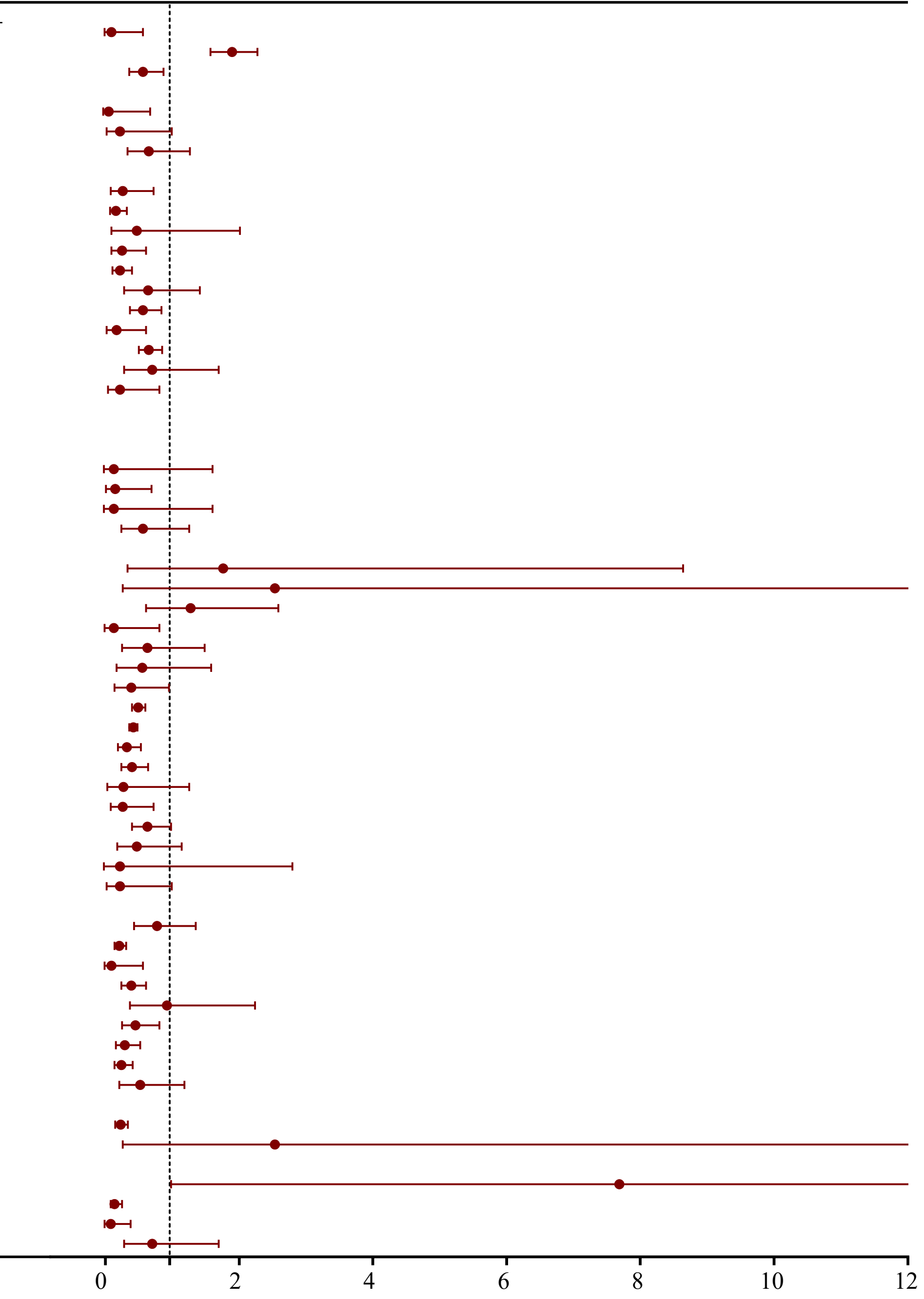

PT	Male/Female	ROR(95%CI)
acute generalised exanthematous pustulosis	8/2	7.8 (1.66 - 36.72)
alopecia	151/566	0.52 (0.43 - 0.62)
blister	41/48	1.67 (1.1 - 2.53)
cutaneous lupus erythematosus	0/3	\
cutaneous symptom	6/1	11.7 (1.41 - 97.15)
decubitus ulcer	6/3	3.9 (0.97 - 15.59)
dermatitis	17/23	1.44 (0.77 - 2.7)
dermatitis acneiform	0/11	\
dermatitis allergic	12/7	3.34 (1.32 - 8.49)
dermatitis bullous	39/15	5.07 (2.8 - 9.2)
dermatitis exfoliative	4/4	1.95 (0.49 - 7.79)
dermatitis exfoliative generalised	16/9	3.47 (1.53 - 7.84)
drug eruption	38/19	3.9 (2.25 - 6.77)
drug reaction with eosinophilia and systemic symptoms	12/16	1.46 (0.69 - 3.09)
dry skin	48/56	1.67 (1.14 - 2.46)
epidermal necrosis	10/4	4.87 (1.53 - 15.54)
erythema	103/139	1.45 (1.12 - 1.87)
erythema multiforme	9/13	1.35 (0.58 - 3.16)
exfoliative rash	8/4	3.9 (1.17 - 12.95)
hair growth abnormal	0/12	\
henoch-schonlein purpura	0/4	\
intertrigo	3/0	\
leukoderma	3/1	5.85 (0.61 - 56.22)
lichenoid keratosis	8/3	5.2 (1.38 - 19.6)
lymphomatoid papulosis	3/1	5.85 (0.61 - 56.22)
night sweats	12/14	1.67 (0.77 - 3.61)
onychoclasis	0/11	
onycholysis	2/7	0.56 (0.12 - 2.68)
onychomadesis	1/5	0.39 (0.05 - 3.34)
palmar-plantar erythrodysaesthesia syndrome	11/28	0.77 (0.38 - 1.54)
pemphigoid	6/2	5.85 (1.18 - 28.98)
petechiae	10/13	1.5 (0.66 - 3.42)
photosensitivity reaction	7/8	1.71 (0.62 - 4.7)
pigmentation disorder	12/10	2.34 (1.01 - 5.41)
pruritus	208/216	1.88 (1.56 - 2.28)
rash	464/418	2.18 (1.91 - 2.49)
rash erythematous	44/31	2.77 (1.75 - 4.38)
rash maculo-papular	43/37	2.27 (1.46 - 3.52)
rash morbilliform	5/3	3.25 (0.78 - 13.59)
rash papular	12/7	3.34 (1.32 - 8.49)
rash pruritic	39/51	1.49 (0.98 - 2.26)
rash vesicular	11/11	1.95 (0.85 - 4.5)
severe cutaneous adverse reaction	2/1	3.9 (0.35 - 42.99)
sjs-ten overlap	6/3	3.9 (0.97 - 15.59)
skin discharge	3/0	\
skin discolouration	21/33	1.24 (0.72 - 2.14)
skin disorder	109/54	3.94 (2.85 - 5.47)
skin erosion	8/2	7.8 (1.66 - 36.72)
skin exfoliation	48/40	2.34 (1.54 - 3.56)
skin hyperpigmentation	8/15	1.04 (0.44 - 2.45)
skin lesion	27/26	2.02 (1.18 - 3.47)
skin reaction	37/24	3.01 (1.8 - 5.03)
skin toxicity	51/28	3.55 (2.24 - 5.64)
skin ulcer	12/13	1.8 (0.82 - 3.94)
spider naevus	0/18	\
stevens-johnson syndrome	88/46	3.74 (2.61 - 5.34)
subcutaneous emphysema	1/5	0.39 (0.05 - 3.34)
symmetrical drug-related intertriginous and flexural exanthema	9/0	\
telangiectasia	1/15	0.13 (0.02 - 0.98)
toxic epidermal necrolysis	67/24	5.45 (3.42 - 8.69)
toxic erythema of chemotherapy	13/3	8.45 (2.41 - 29.65)
toxic skin eruption	9/13	1.35 (0.58 - 3.16)

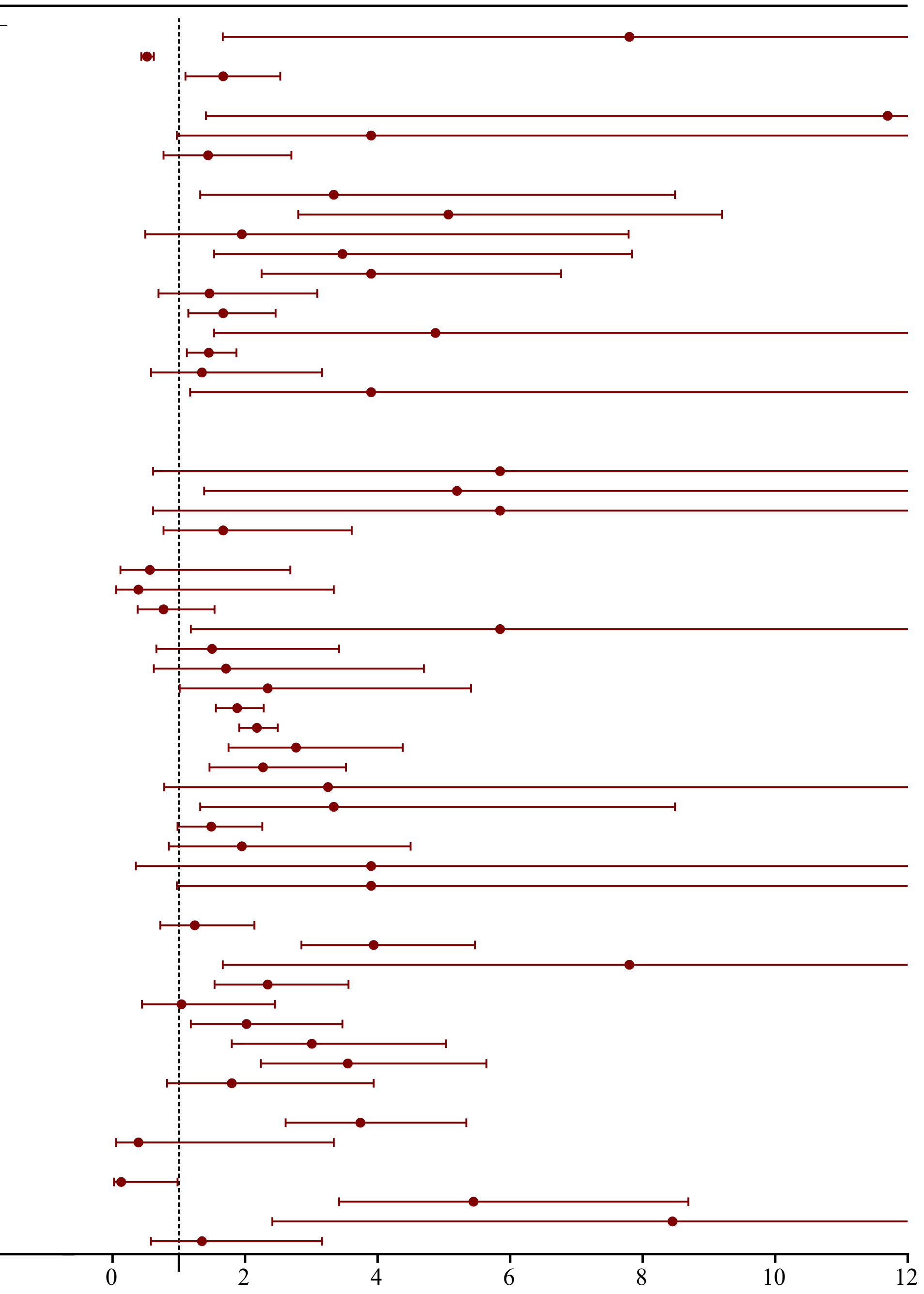

Supplementary Figure 3. Forest plot of head-to-head subgroup analysis of ADC-associated CAEs stratified by gender. From left to right, PT level, gender subgroup, ROR values with confidence intervals, and the corresponding forest plots are presented.
