# Supplementary material for: Cutaneous adverse events with antibody-drug conjugates: a FAERS-based pharmacovigilance study
Source: Front Med (Lausanne). 2026 May 25;13:1847032. doi: 10.3389/fmed.2026.1847032 (PMC13243105; doi:10.3389/fmed.2026.1847032)
Supplement: Supplementary file 5 [file Data_Sheet_4.pdf]

PT	Young/Elderly	ROR(95%Cl)
acute generalised exanthematous pustulosis	2/6	0.23 (0.05 - 1.15)
alopecia	242/229	0.73 (0.61 - 0.88)
blister	25/39	0.45 (0.27 - 0.74)
cutaneous lupus erythematosus	2/1	1.39 (0.13 - 15.33)
cutaneous symptom	2/4	0.35 (0.06 - 1.9)
decubitus ulcer	2/5	0.28 (0.05 - 1.43)
dermatitis	15/17	0.61 (0.31 - 1.23)
dermatitis acneiform	7/0	\
dermatitis allergic	12/6	1.39 (0.52 - 3.7)
dermatitis bullous	14/30	0.32 (0.17 - 0.61)
dermatitis exfoliative	3/3	0.69 (0.14 - 3.44)
dermatitis exfoliative generalised	10/14	0.5 (0.22 - 1.12)
drug eruption	16/39	0.28 (0.16 - 0.51)
drug reaction with eosinophilia and systemic symptoms	15/12	0.87 (0.41 - 1.86)
dry skin	38/47	0.56 (0.37 - 0.86)
epidermal necrosis	7/5	0.97 (0.31 - 3.07)
erythema	89/101	0.61 (0.46 - 0.81)
erythema multiforme	5/14	0.25 (0.09 - 0.69)
exfoliative rash	2/10	0.14 (0.03 - 0.63)
hair growth abnormal	3/2	1.04 (0.17 - 6.24)
henoch-schonlein purpura	1/2	0.35 (0.03 - 3.83)
intertrigo	0/2	\
leukoderma	1/3	0.23 (0.02 - 2.23)
lichenoid keratosis	3/6	0.35 (0.09 - 1.39)
lymphomatoid papulosis	4/0	\
night sweats	21/2	7.3 (1.71 - 31.14)
onychoclasis	5/2	1.74 (0.34 - 8.96)
onycholysis	1/1	0.69 (0.04 - 11.11)
onychomadesis	3/2	1.04 (0.17 - 6.24)
palmar-plantar erythrodysaesthesia syndrome	18/12	1.04 (0.5 - 2.16)
pemphigoid	0/7	\
petechiae	14/7	1.39 (0.56 - 3.44)
photosensitivity reaction	3/3	0.69 (0.14 - 3.44)
pigmentation disorder	4/13	0.21 (0.07 - 0.66)
pruritus	145/207	0.48 (0.39 - 0.6)
rash	234/365	0.44 (0.37 - 0.52)
rash erythematous	23/33	0.48 (0.28 - 0.82)
rash maculo-papular	38/34	0.78 (0.49 - 1.23)
rash morbilliform	4/2	1.39 (0.25 - 7.59)
rash papular	2/14	0.1 (0.02 - 0.44)
rash pruritic	30/44	0.47 (0.3 - 0.75)
rash vesicular	1/14	0.05 (0.01 - 0.38)
severe cutaneous adverse reaction	1/2	0.35 (0.03 - 3.83)
sjs-ten overlap	0/7	\
skin discharge	3/0	\
skin discolouration	13/18	0.5 (0.25 - 1.02)
skin disorder	25/124	0.14 (0.09 - 0.21)
skin erosion	4/6	0.46 (0.13 - 1.64)
skin exfoliation	22/43	0.36 (0.21 - 0.59)
skin hyperpigmentation	7/3	1.62 (0.42 - 6.27)
skin lesion	22/11	1.39 (0.67 - 2.87)
skin reaction	17/15	0.79 (0.39 - 1.58)
skin toxicity	14/42	0.23 (0.13 - 0.42)
skin ulcer	9/10	0.63 (0.25 - 1.54)
spider naevus	11/1	7.65 (0.99 - 59.23)
stevens-johnson syndrome	22/71	0.21 (0.13 - 0.35)
subcutaneous emphysema	5/0	\
symmetrical drug-related intertriginous and flexural exanthema	1/8	0.09 (0.01 - 0.69)
telangiectasia	12/0	\
toxic epidermal necrolysis	14/66	0.15 (0.08 - 0.26)
toxic erythema of chemotherapy	6/10	0.42 (0.15 - 1.15)
toxic skin eruption	9/9	0.69 (0.28 - 1.75)

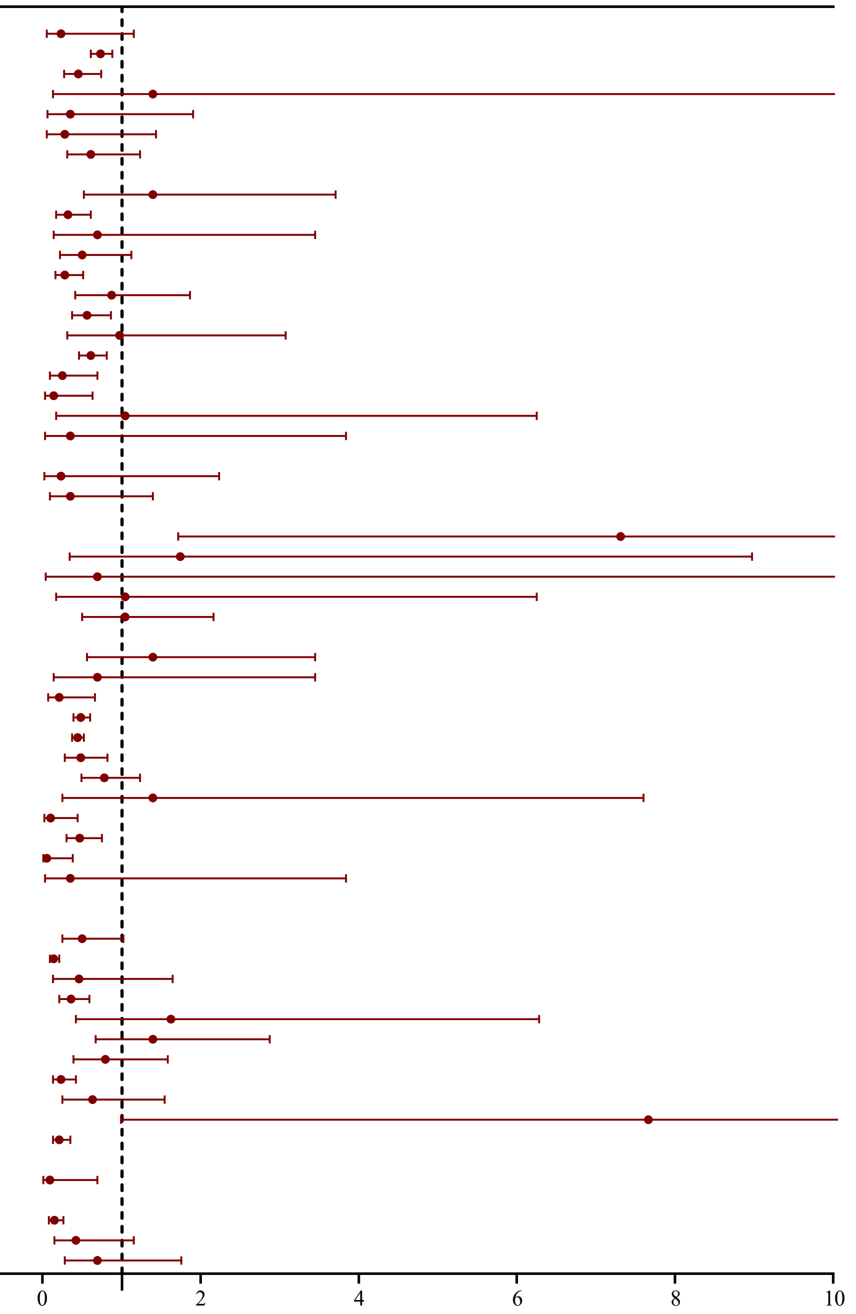

PT	Elderly/Young	ROR(95%Cl)
acute generalised exanthematous pustulosis	6/2	4.32 (0.87 - 21.39)
alopecia	229/242	1.36 (1.14 - 1.64)
blister	39/25	2.25 (1.36 - 3.71)
cutaneous lupus erythematosus	1/2	0.72 (0.07 - 7.93)
cutaneous symptom	4/2	2.88 (0.53 - 15.71)
decubitus ulcer	5/2	3.6 (0.7 - 18.55)
dermatitis	17/15	1.63 (0.81 - 3.27)
dermatitis acneiform	0/7	\
dermatitis allergic	6/12	0.72 (0.27 - 1.92)
dermatitis bullous	30/14	3.09 (1.64 - 5.82)
dermatitis exfoliative	3/3	1.44 (0.29 - 7.13)
dermatitis exfoliative generalised	14/10	2.01 (0.89 - 4.54)
drug eruption	39/16	3.51 (1.96 - 6.28)
drug reaction with eosinophilia and systemic symptoms	12/15	1.15 (0.54 - 2.46)
dry skin	47/38	1.78 (1.16 - 2.73)
epidermal necrosis	5/7	1.03 (0.33 - 3.24)
erythema	101/89	1.64 (1.23 - 2.18)
erythema multiforme	14/5	4.03 (1.45 - 11.19)
exfoliative rash	10/2	7.2 (1.58 - 32.85)
hair growth abnormal	2/3	0.96 (0.16 - 5.74)
henoch-schonlein purpura	2/1	2.88 (0.26 - 31.74)
intertrigo	2/0	\
leukoderma	3/1	4.32 (0.45 - 41.51)
lichenoid keratosis	6/3	2.88 (0.72 - 11.51)
lymphomatoid papulosis	0/4	\
night sweats	2/21	0.14 (0.03 - 0.58)
onychoclasis	2/5	0.58 (0.11 - 2.97)
onycholysis	1/1	1.44 (0.09 - 23.01)
onychomadesis	2/3	0.96 (0.16 - 5.74)
palmar-plantar erythrodysaesthesia syndrome	12/18	0.96 (0.46 - 1.99)
pemphigoid	7/0	\
petechiae	7/14	0.72 (0.29 - 1.78)
photosensitivity reaction	3/3	1.44 (0.29 - 7.13)
pigmentation disorder	13/4	4.68 (1.53 - 14.35)
pruritus	207/145	2.06 (1.67 - 2.55)
rash	365/234	2.26 (1.92 - 2.67)
rash erythematous	33/23	2.07 (1.21 - 3.52)
rash maculo-papular	34/38	1.29 (0.81 - 2.05)
rash morbilliform	2/4	0.72 (0.13 - 3.93)
rash papular	14/2	10.08 (2.29 - 44.34)
rash pruritic	44/30	2.11 (1.33 - 3.36)
rash vesicular	14/1	20.15 (2.65 - 153.28)
severe cutaneous adverse reaction	2/1	2.88 (0.26 - 31.74)
sjs-ten overlap	7/0	\
skin discharge	0/3	\
skin discolouration	18/13	1.99 (0.98 - 4.07)
skin disorder	124/25	7.17 (4.66 - 11.01)
skin erosion	6/4	2.16 (0.61 - 7.65)
skin exfoliation	43/22	2.82 (1.68 - 4.71)
skin hyperpigmentation	3/7	0.62 (0.16 - 2.38)
skin lesion	11/22	0.72 (0.35 - 1.48)
skin reaction	15/17	1.27 (0.63 - 2.54)
skin toxicity	42/14	4.32 (2.36 - 7.91)
skin ulcer	10/9	1.6 (0.65 - 3.94)
spider naevus	1/11	0.13 (0.02 - 1.01)
stevens-johnson syndrome	71/22	4.65 (2.88 - 7.51)
subcutaneous emphysema	0/5	\
symmetrical drug-related intertriginous and flexural exanthema	8/1	11.51 (1.44 - 92.07)
telangiectasia	0/12	\
toxic epidermal necrolysis	66/14	6.8 (3.82 - 12.1)
toxic erythema of chemotherapy	10/6	2.4 (0.87 - 6.6)
toxic skin eruption	9/9	1.44 (0.57 - 3.63)

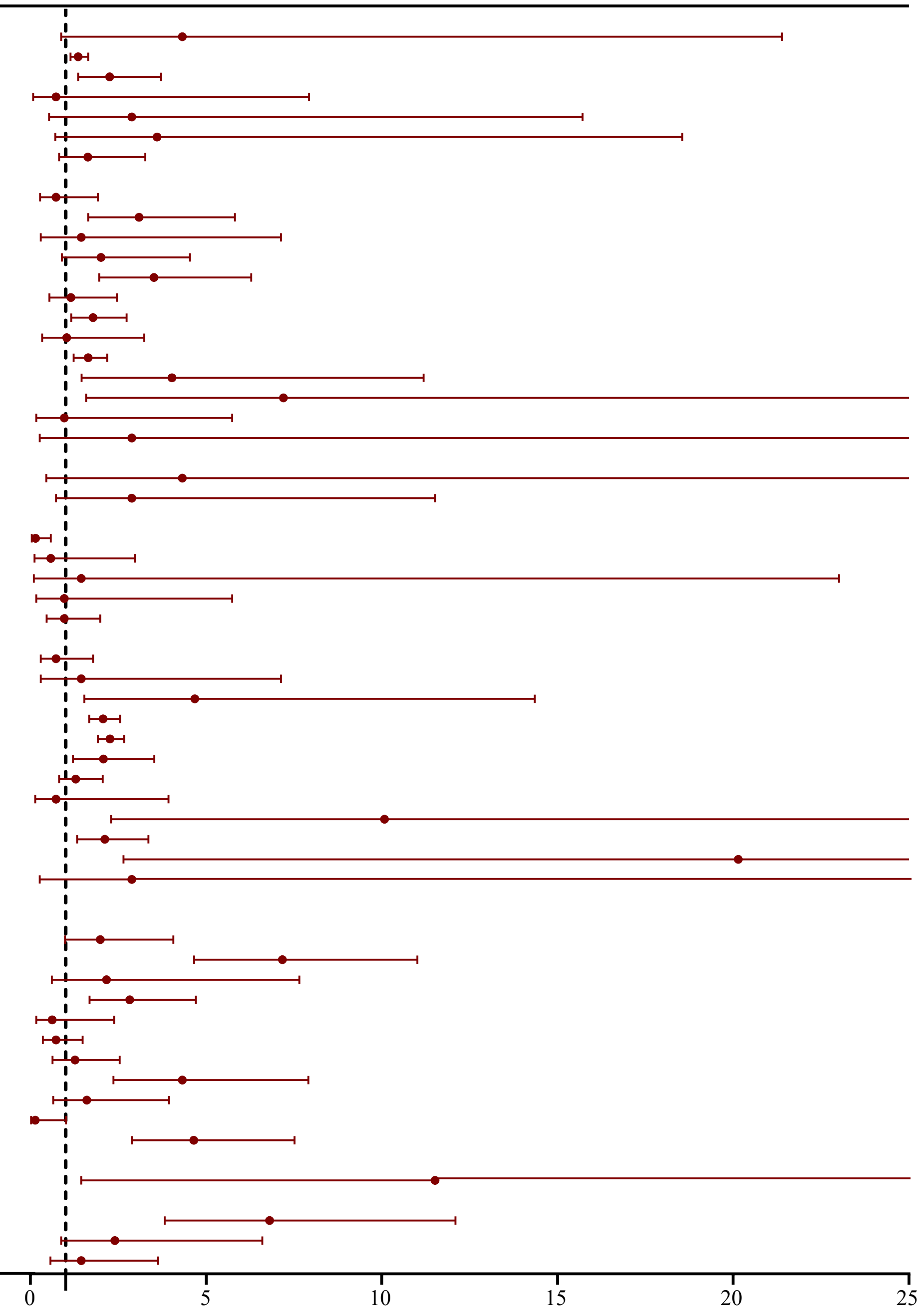

Supplementary Figure 4. Forest plot of head-to-head subgroup analysis of ADC-associated CAEs stratified by age. From left to right, PT level, gender subgroup, ROR values with confidence intervals, and the corresponding forest plots are presented.
